# Cordycepin Targets HRD1 to Promote Cancer Cell PD‐L1 Ubiquitin–Proteasome Degradation and Increase Antitumor Immunity

**DOI:** 10.1002/mco2.70430

**Published:** 2025-10-17

**Authors:** Xiangxin Geng, Minchen Cai, Hongmei Hu, Mengting Xu, Qing Zhang, Hanchen Xu, Dianping Yu, Hongwei Zhang, Hanchi Xu, Linyang Li, Mengmeng Guo, Shize Xie, Qun Wang, Weidong Zhang, Sanhong Liu

**Affiliations:** ^1^ State Key Laboratory of Discovery and Utilization of Functional Components in Traditional Chinese Medicine Shanghai Frontiers Science Center of TCM Chemical Biology Institute of Interdisciplinary Integrative Medicine Research Shanghai University of Traditional Chinese Medicine Shanghai China; ^2^ Institute of Digestive Diseases Longhua Hospital Shanghai University of Traditional Chinese Medicine Shanghai China; ^3^ Department of Phytochemistry School of Pharmacy Second Military Medical University Shanghai China; ^4^ Institute of Medicinal Plant Development Chinese Academy of Medical Sciences and Peking Union Medical College Beijing China; ^5^ The Research Center For Traditional Chinese Medicine Shanghai Institute of Infectious Diseases and Biosafety Institute of Interdisciplinary Integrative Medicine Research Shanghai University of Traditional Chinese Medicine Shanghai China

**Keywords:** *Cordyceps militaris* extract (CME), cordycepin (COR), colorectal cancer (CRC), HMG‐CoA reductase degradation protein 1 (HRD1), programmed death‐ligand 1 (PD‐L1)

## Abstract

Immune checkpoint blockade has become an effective strategy for inhibiting tumor growth, especially immune checkpoint inhibitors that target the programmed death 1 (PD‐1)/programmed death‐ligand 1 (PD‐L1) pathway, which have shown significant effects in tumor immunotherapy. In this study, we found that naturally sourced *Cordyceps militaris* extract can effectively downregulate the protein expression level of PD‐L1 in human colorectal cancer cell lines. Further systematic isolation, purification, and analysis of its active components revealed that cordycepin (COR) is the key active molecule mediating PD‐L1 degradation. Mechanistically, COR specifically and selectively targets the ubiquitin E3 ligase HMG‐CoA reductase degradation protein 1, thus promoting the degradation of PD‐L1 protein through the ubiquitin–proteasome pathway. This process significantly enhances the cytotoxic killing effect of effector T lymphocytes against colorectal cancer cells, ultimately achieving robust antitumor effects. Furthermore, this study also revealed that COR exhibits potential synergistic therapeutic effects when combined with anti‐CTLA4 antibodies in preclinical tumor treatment. In summary, COR, as the primary bioactive component of *Cordyceps* *militaris*, demonstrates considerable potential to act as a small‐molecule immune checkpoint modulator and inhibitor, thereby providing a novel therapeutic strategy for the immunotherapy of colorectal cancer.

## Graphical abstract

Using an activity‐based tracking approach, our study revealed that the component in *Cordyceps militaris* extract (CME) that reduces PD‐L1 activity is cordycepin, which demonstrates potential as an immune checkpoint inhibitor for colorectal cancer. Cordycepin targets HRD1, leading to PD‐L1 protein degradation via the ubiquitin–proteasome pathway, which enhances T cell cytotoxicity against cancer cells.

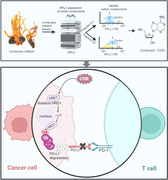

## 1 Introduction

Colorectal cancer (CRC) is a globally prevalent malignancy with significant incidence and mortality rates [1]. According to the latest statistics, there are over 1 million new cases globally each year, with nearly 1 million deaths. Currently, the treatment of CRC relies mainly on traditional methods such as surgery, chemotherapy, radiotherapy, and targeted therapy [2]. Although these treatment methods have extended the survival time of patients to some extent, due to drug resistance, severe side effects, and posttreatment recurrence and metastasis, the therapeutic effect is not ideal.

The swift advancement in tumor immunotherapy has ushered in novel therapeutic approaches for CRC. Notably, anti‐PD‐1 therapies have proven effective by blocking the interaction between PD‐1 and its ligand PD‐L1, thereby revolutionizing CRC treatment. PD‐L1 plays a pivotal role in immune suppression; it binds to PD‐1 within the tumor microenvironment, inhibiting T cell activity and enabling tumor cells to evade immune detection [3, 4, 5]. Anti‐PD‐1 therapies, which block PD‐1/PD‐L1 binding, have proven highly effective in treating various cancers by reactivating the patient's immune system. However, PD‐1 antibody therapy also has certain limitations, such as limitations in terms of administration methods [6], potential immune‐related adverse reactions, and intrinsic or acquired resistance [7], which restrict its wide application in clinical practice.

Compared with PD‐1 antibodies, small‐molecule compounds have unique advantages in regulating PD‐L1 expression [8]. Small molecule compounds can reduce the expression of PD‐L1 through different mechanisms, such as interference with the transcription, translation, or posttranslational modification of PD‐L1. For instance, PD‐L1 expression is reduced by zoledronic acid triggering its autophagic breakdown [9], thereby enhancing the antitumor capabilities of the immune system. This method of reducing PD‐L1 via the ubiquitin–proteasome pathway not only lowers PD‐L1 levels on tumor cells, restoring the ability of the immune system to recognize and eliminate them, but also offers advantages over antibody drugs. Small molecule compounds are often more orally bioavailable, cost effective, and easier to transport, suggesting broader clinical potential. Thus, developing small molecules that regulate PD‐L1 expression, particularly those inducing its degradation, may be a future direction for CRC immunotherapy [10, 11]. These compounds could address PD‐1 antibody therapy limitations and provide more effective, safer, and affordable options for patients. Future work should explore their mechanisms, refine treatment protocols, and validate them clinically to enhance therapeutic outcomes for patients with CRC.

*Cordyceps militaris* extract (CME), commonly referred to as cordyceps, is a highly prized medicinal fungus in traditional Chinese medicine. It comprises numerous bioactive substances, such as cordycepin (COR), COR acid, cordyceps polysaccharides, and selenium. These components endow cordyceps with various pharmacological activities, including antibacterial properties [12], antiviral effects, prevention and treatment of cerebral thrombosis, and enhancement of human immunity [13]. Our research revealed that *Cordyceps* *militaris* can also regulate the expression of the key protein PD‐L1 in tumor immune escape. Further active tracking revealed that, compared with traditional agents, such as those with anti‐inflammatory, antibacterial, and antiviral effects, COR is the active component that regulates PD‐L1 expression. Mechanistically, COR triggers PD‐L1 protein degradation via the ubiquitin–proteasome pathway by targeting HMG‐CoA reductase degradation protein 1 (HRD1), enhancing antitumor immunity. These findings offer new theoretical support for the use of COR in tumor immunotherapy and suggest a novel immunotherapy strategy.

## 2 Results

### 2.1 CME Negatively Regulates the Expression of PD‐L1 in Tumor Cells

**FIGURE 1:**
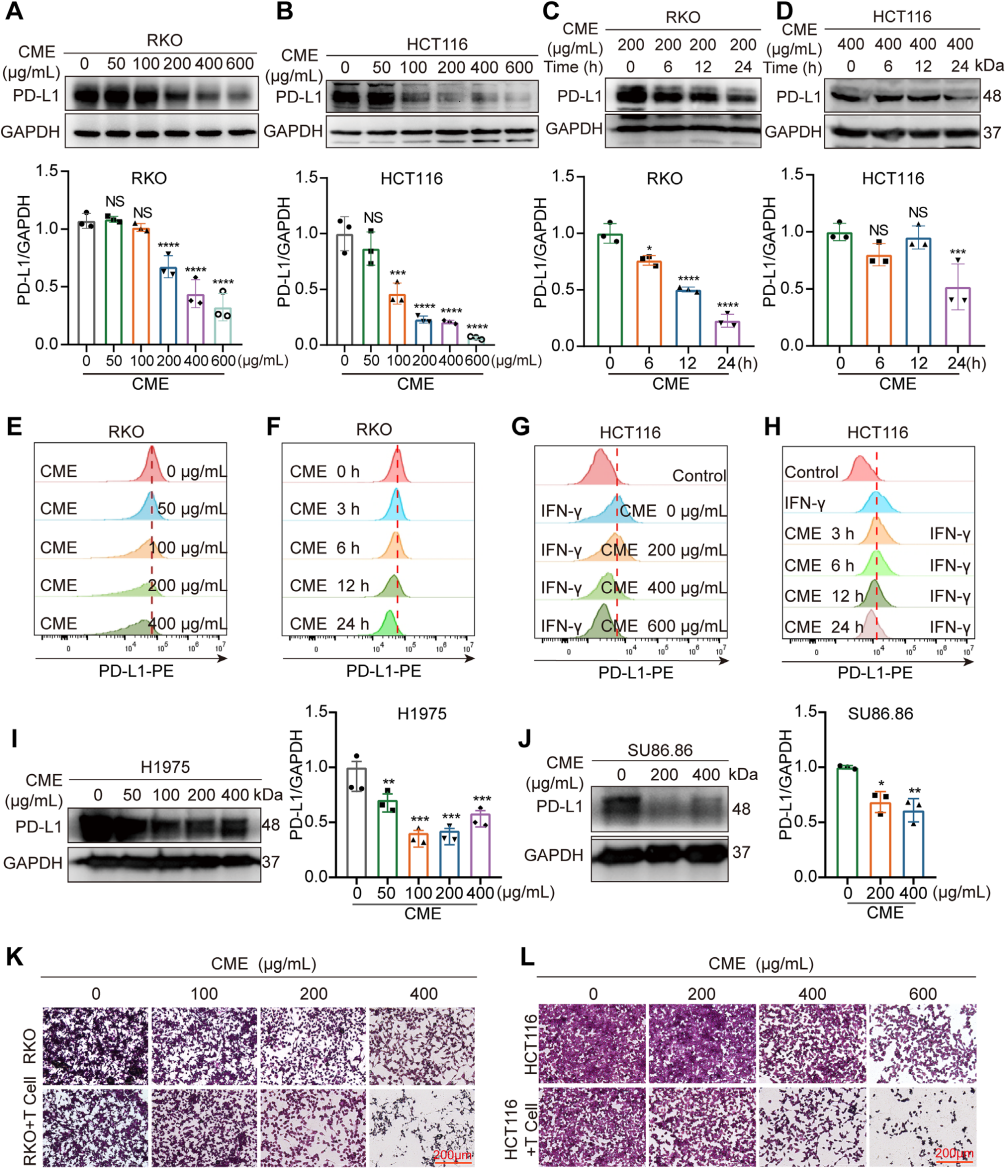
Effect of CME on PD‐L1 degradation in tumor cells. (A and B) PD‐L1 expression in HCT116 and RKO cells after 24 h of CME treatment was analyzed by Western blotting. (C and D) Western blot analysis of PD‐L1 levels in RKO cells exposed to 200 µg/mL CME for various durations and in HCT116 cells subjected to 400 µg/mL CME for different durations. (E and F) Flow cytometric analysis was employed to assess PD‐L1 levels in RKO cells after exposure to various concentrations of CME for 24 h or to 200 µg/mL CME for different time periods. (G and H) After IFN‐γ stimulation, HCT116 cells presented increased PD‐L1 expression on their cell membrane surface. Flow cytometry was then used to detect changes in PD‐L1 levels in HCT116 cells following treatment with various concentrations of CME for 24 h or with 400 µg/mL CME for different durations. (I and J) PD‐L1 levels in the human lung cancer cell line H1975 and the human pancreatic cancer cell line SU86.86 were detected via Western blotting after 24 h of treatment with different concentrations of CME. (K and L) PD‐1‐overexpressing Jurkat cells were cocultured with RKO and HCT116 cells and subjected to varying concentrations of CME for 24 h, after which tumor cell survival was determined through crystal violet staining. We present the data as the mean ± SEM, with statistical significance evaluated via Student's *t*‐test. *p < 0.05, **p < 0.01, ***p < 0.001, and ****p < 0.0001; NS, not significant.

To explore how CME influences PD‐L1 expression in CRC cells, we conducted Western blotting to measure overall PD‐L1 protein levels and flow cytometry to analyze PD‐L1 protein levels on the cell membrane. Our findings revealed that CME notably decreased both total and membrane‐associated PD‐L1 protein levels in HCT116 and RKO cells. Moreover, this reduction was observed to be both concentration dependent and time dependent (Figures 1A–H and S1A–D). We subsequently conducted immunofluorescence experiments to further investigate the regulatory effect of CME on the expression of membranous PD‐L1. The immunofluorescence results demonstrated that CME significantly reduced the level of PD‐L1 on the surface of RKO and HCT116 cells (Figure S1E–H), further indicating that CME can decrease the translocation of PD‐L1 to the plasma membrane. To further explore the capacity of CME to reduce PD‐L1 expression, we conducted experiments on additional cancer cell lines, including the H1975 lung cancer cell line and the SU86.86 pancreatic cancer cell line, and used immunoblotting (IB) to measure PD‐L1 expression levels. Our data indicate that treatment with CME leads to a significant and dose‐dependent reduction in PD‐L1 expression across these cell lines (Figure 1I,1J). This consistent reduction in PD‐L1 expression suggests that CME may have a broad‐spectrum effect on PD‐L1 downregulation in various cancer types.

Additionally, to evaluate the direct cytotoxic effects of CME, we conducted CCK‐8 and EdU assays on the RKO, HCT116, and normal NCM460 cell lines. Although CME exhibited some cytotoxicity, its inhibitory effect on cell proliferation was not significant (Figure S2A–D). The results showed that 200 µg/mL is a safe and effective dose of CME for studying PD‐L1 downregulation. Given that PD‐L1 on tumor cells inhibits T‐cell activity by binding PD‐1, we investigated whether CME could restore antitumor immunity [14]. Then, we cocultured RKO or HCT116 cells with PD‐1‐overexpressing Jurkat cells and assessed tumor cell survival via crystal violet staining. Compared with the control, CME treatment significantly reduced RKO cell survival, indicating that CME can increase T cell cytotoxicity toward cancer cells by downregulating PD‐L1 (Figures 1K,1L and S2E,F).

### 2.2 COR is the Main Component of CME that Negatively Regulates the Expression of PD‐L1 in CRC Cells

**FIGURE 2:**
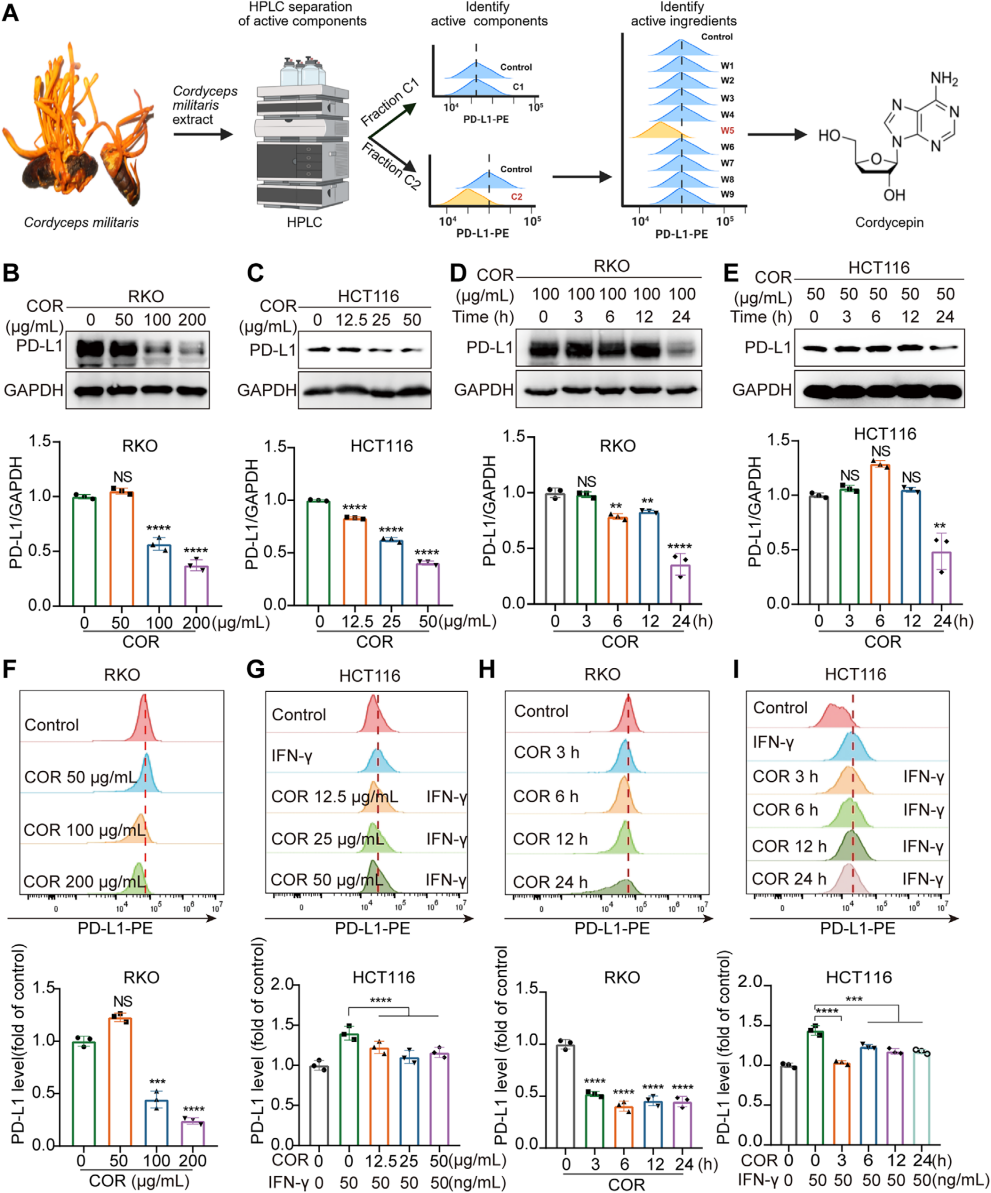
COR negatively regulates PD‐L1 in colorectal cancer cells. (A) Isolation and confirmation of PD‐L1‐reducing active ingredients in *Cordyceps militaris*. (B and C) Western blotting was used to assess PD‐L1 levels in RKO and HCT116 cells after 24 h of exposure to various doses of COR. (D and E) PD‐L1 levels in RKO cells treated with 100 µg/mL COR for different durations and in HCT116 cells treated with 50 µg/mL COR for various durations were analyzed via Western blotting. (F and G) Flow cytometry was used to evaluate the impact of treating RKO cells with assorted concentrations of COR for 24 h and stimulating HCT116 cells with IFN‐γ on PD‐L1 cell membrane expression. (H and I) The effects of COR on PD‐L1 cell membrane expression in IFN‐γ‐stimulated RKO and HCT116 cells over different time intervals were also assessed via flow cytometry. Statistical significance is indicated as *p < 0.05, **p < 0.01, ***p < 0.001, and ****p < 0.0001.

In our search for the active component that impacts PD‐L1 expression in tumor cells, we used high‐performance liquid chromatography to fractionate the CME extract and successfully isolated two primary fractions, namely, C1 and C2. Subsequent flow cytometry analysis revealed that the C2 fraction possesses effective components capable of downregulating PD‐L1 expression. Further separation divided C2 into several parts, W1 to W9, among which the W5 fraction, owing to its high content of COR, significantly downregulated PD‐L1 expression (Figures 2A and S3A–D). These findings indicate that COR may be a key active component in CME that regulates PD‐L1 expression.

To further verify whether COR is the active ingredient in CME that suppresses PD‐L1 expression, Western blotting was performed for validation. The results revealed that COR substantially reduced PD‐L1 levels in RKO and HCT116 cells in a dose‐ and time‐dependent manner (Figure 2B–E), which was confirmed by flow cytometry (Figure 2F–I). Additionally, immunofluorescence experiments further demonstrated that COR reduced PD‐L1 protein expression on the surface of RKO and HCT116 cells (Figure S3E,F). Furthermore, CCK‐8 assays revealed that within the effective concentration range, COR did not have significant cytotoxic effects on normal intestinal epithelium (NCM460) or CRC cells (Figure S3G–I). In summary, we speculate that COR is the active component of CME that reduces the expression of PD‐L1 in CRC cells.

### 2.3 COR Promotes T Cell Activation and T Cell‐Mediated Killing of Cancer Cells in Vitro and in Vivo

**FIGURE 3:**
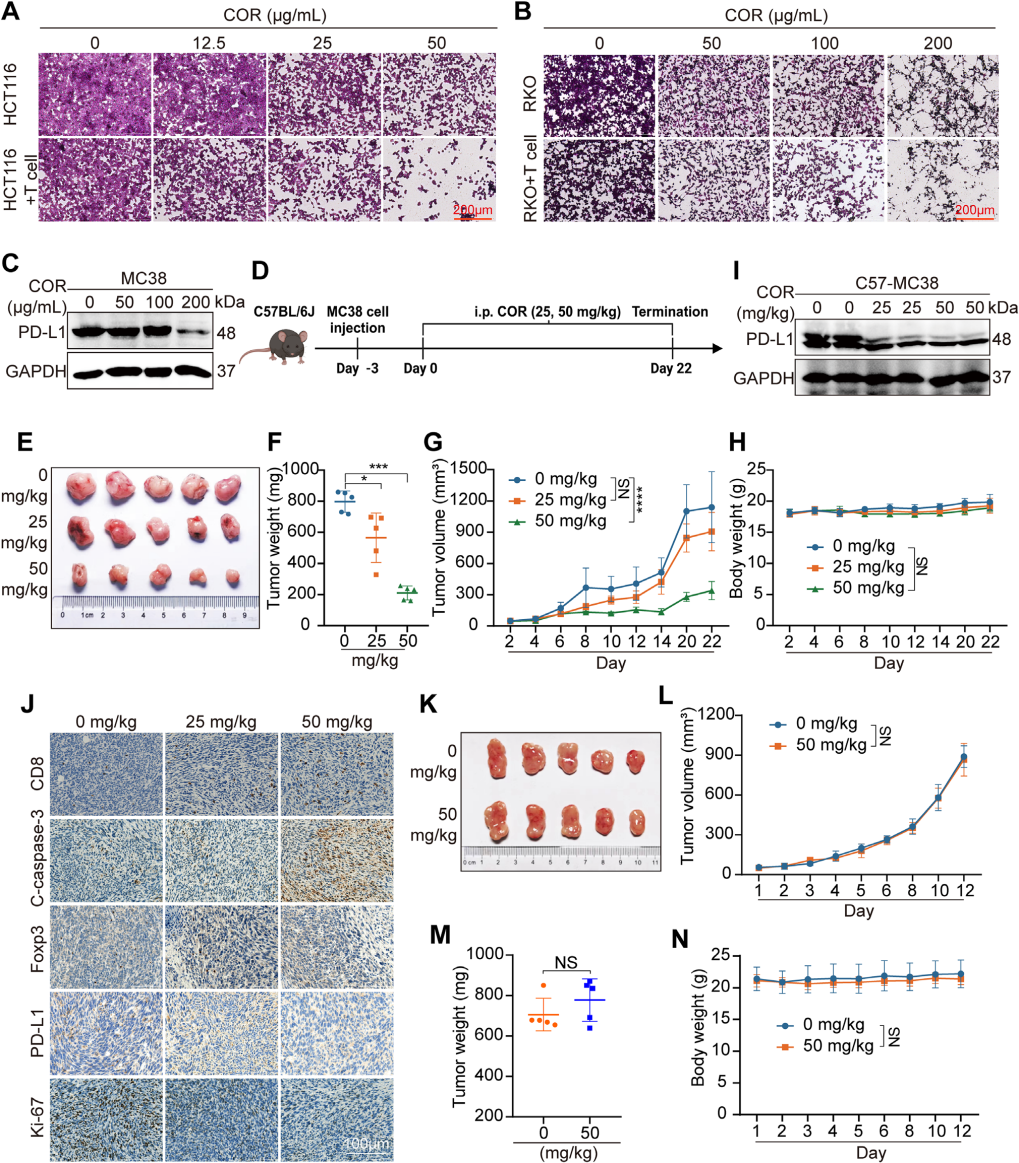
COR enhances the killing ability of T cells and exerts antitumor effects in vivo through T cells. (A and B) Jurkat cells were cultured alongside HCT116 and RKO cells and subjected to different concentrations of COR for 24 h. Subsequently, tumor cell viability was evaluated via crystal violet staining, with a scale of 200 µm. (C) Western blotting was used to investigate how COR affects PD‐L1 expression in MC38 cells. (D) Schematic diagram related to animal experimentation on female C57BL/6J mice. (E) Visual image of solid tumors in C57BL/6J mice from diverse treatment cohorts. (F) Comparative analysis of tumor weight in C57BL/6J mice receiving different treatments. (G) Growth curves of mouse tumors during drug administration. (H) Weight statistics of tumor‐bearing mice in different treatment groups. (I) Western blotting was used to track fluctuations in PD‐L1 expression within the tumor tissues of mice subjected to COR treatment. (J) Results of IHC staining for CD8, cleaved caspase‐3, Foxp3, PD‐L1, and Ki‐67 in C57BL/6J mouse tumors from the control group or COR. (Scale bar = 100 µm). (K) Comparison of solid tumor photos between immunocompromised mice in the treatment group and the control group. (L) Growth curves of the nude mouse tumors. (M) Comparative analysis of tumor weight in nude mice. (N) Statistical analysis of the weights of the tumor‐bearing mice. The data are expressed as the mean ± SEM, and Student's *t*‐test was used to evaluate statistical significance. The notation forstatistical significance is defined as *p < 0.05, **p < 0.01, ***p < 0.001, and ****p < 0.0001.

To evaluate the in vitro antitumor effects of COR, we conducted coculture experiments with RKO or HCT116 cells and Jurkat cells overexpressing PD‐1 and assessed tumor cell survival via crystal violet staining. The results demonstrated that COR reduces tumor cell survival, indicating that it can enhance T cell cytotoxicity against these cells (Figures 3A,B and S4A,B). To further explore the in vivo antitumor effects of COR, we first examined its impact on PD‐L1 expression in MC38 cells and observed that COR downregulated PD‐L1 (Figure 3C). We subsequently used the mouse‐derived CRC cell line MC38 as a model for subsequent experiments. MC38 cells were subcutaneously injected into female C57BL/6J mice to establish a subcutaneous CRC model. The experimental mice were subsequently divided into three groups: a control group, a 25 mg/kg dosage group, and a 50 mg/kg dosage group (Figure 3D). Thereafter, the mice were administered the solvent or COR via intraperitoneal injection to explore the in vivo antitumor effects of COR. Statistical analysis of the tumor weights and volumes in the mice revealed that COR treatment significantly suppressed MC38 tumor growth, resulting in inhibition rates of 20.49 and 70.11% at doses of 25 and 50 mg/kg, respectively (Figure 3E–G). Moreover, an analysis of the weight changes in the visceral organs and the histological staining results of the mice in the different dosage groups suggested that, at the tested dosages, COR did not cause any significant toxicity in the mice (Figures 3H and S4C).

To further explore the impact of COR on antitumor immune responses in mice, immunohistochemical staining or Western blotting was performed on mouse tumor tissues. The results revealed that COR dose‐dependently increased CD8^+^ T cell and cleaved caspase‐3 expression while decreasing PD‐L1, Foxp3, and Ki‐67 levels (Figures 3I,J and S4D). Notably, COR was ineffective at suppressing MC38 subcutaneous tumor growth in immunodeficient nude mice, highlighting that its antitumor effects are mediated by T cell‐driven immune activation (Figures 3K–N and S4E). In summary, our data suggest that COR exerts significant antitumor effects by enhancing the activity of tumor‐infiltrating T cells.

### 2.4 COR Degrades PD‐L1 Through the Ubiquitination Pathway

**FIGURE 4:**
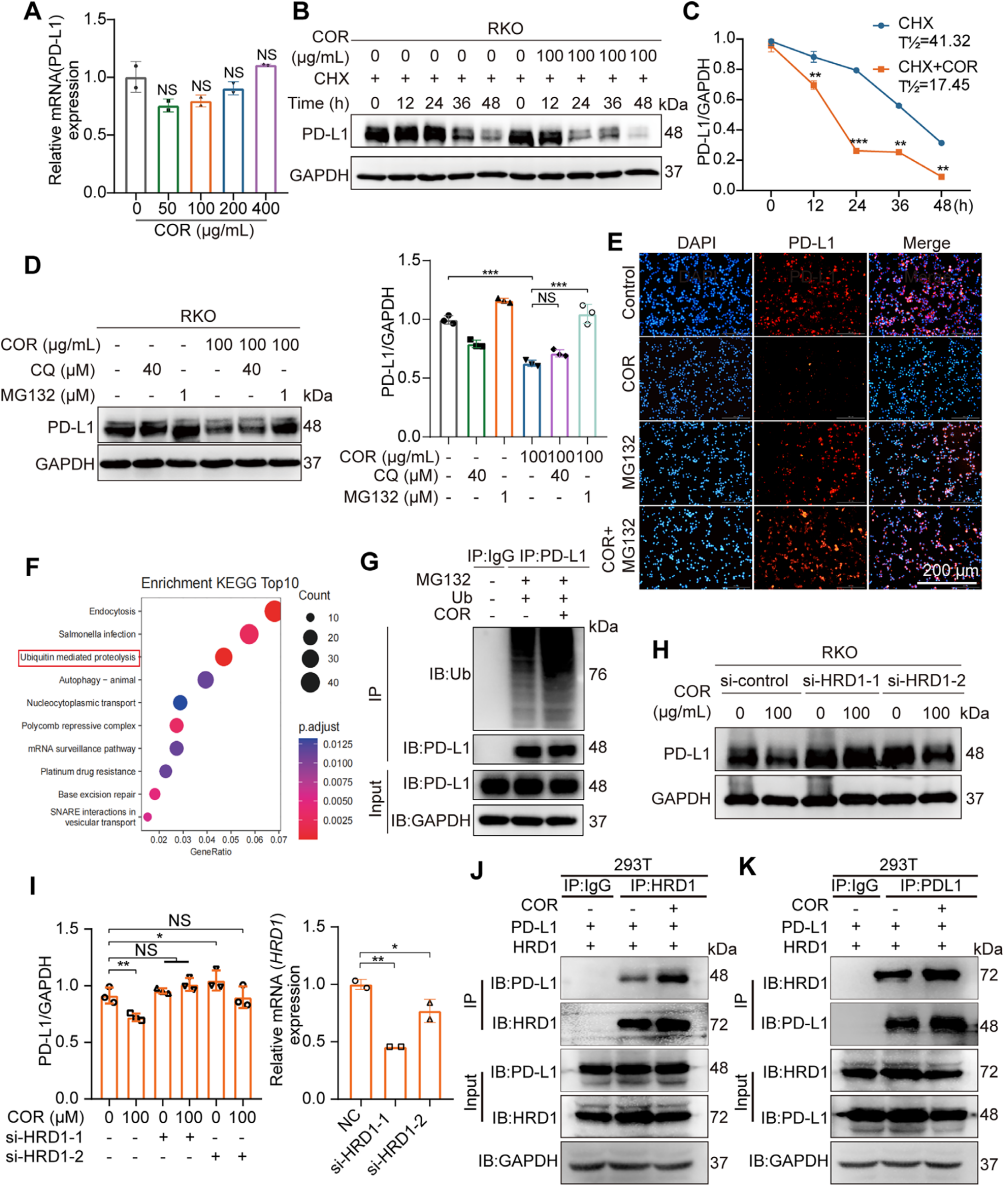
COR through targeting HRD1 promotes the degradation of PD‐L1 via a ubiquitination mechanism. (A) Using quantitative RT‐PCR, we evaluated how different COR concentrations affect PD‐L1 mRNA levels in RKO cells. (B and C) Immunoblotting was used to detect PD‐L1 expression in RKO cells treated with DMSO, 100 µg/mL COR, or cycloheximide (50 mg/mL), and the PD‐L1 protein half‐life was determined. (D) An immunoblot assay was used to measure PD‐L1 expression in RKO cells exposed to COR along with the proteasome inhibitors MG132 and chloroquine. (E) An immunofluorescence assay was used to observe the changes in PD‐L1 protein expression on the surface of RKO cell membranes when COR was used in combination with MG132 or CQ. (F) RKO colorectal cancer cells were incubated with 100 µg/mL COR or PBS for 24 h, after which LC–MS/MS analysis was performed to identify proteins whose expression was altered. (G) RKO cells overexpressing Ub were subjected to immunoprecipitation to assess the degree of PD‐L1 ubiquitination induced by COR. Ubiquitinated PD‐L1 was captured via Flag microbeads and identified via anti‐Ub antibodies. (H and I) After treatment with COR, RKO cells were transfected with HRD1 siRNA or control siRNA, and immunoblotting was subsequently performed to detect PD‐L1. The results showed that HRD1 knockdown abolished COR‐induced PD‐L1 downregulation. (J and K) Co‐IP and IB analysis of 293T cells cotransfected with PD‐L1 and HRD1 plasmids verified their interaction. NS, not significant; *p < 0.05, **p < 0.01, ***p < 0.001, and ****p < 0.0001.

Given that COR substantially decreases PD‐L1 expression in tumor cells, we investigated the underlying regulatory mechanism. Real‐time PCR revealed that PD‐L1 mRNA levels remained relatively stable in RKO cells treated with COR, suggesting that COR has no transcriptional impact (Figure 4A). To assess whether COR triggers PD‐L1 degradation through posttranslational mechanisms, RKO cells were treated with cycloheximide, a protein synthesis inhibitor [15]. COR treatment reduced the half‐life of PD‐L1 and increased its degradation rate (Figure 4B,C). These findings indicate that COR mediates the degradation of PD‐L1 primarily at the protein level without affecting transcription. The PD‐L1 protein undergoes diverse posttranslational modifications, including phosphorylation [16], glycosylation [17], palmitoylation [18], acetylation [19], polyubiquitination [17], and deubiquitination [20]. Such posttranslational modifications can lead to either subcellular relocalization or changes in protein stability. Research has demonstrated that PD‐L1 degradation is predominantly mediated through ubiquitination‐coupled proteasomal pathways and lysosomal degradation systems. These tightly regulated processes play crucial roles in preserving immune homeostasis by suppressing pathological immune evasion [21].

To investigate COR‐induced PD‐L1 degradation, we treated RKO and HCT116 cells with COR plus MG132 (a proteasome inhibitor) or chloroquine (a lysosomal inhibitor). MG132, but not chloroquine, blocked PD‐L1 degradation, suggesting proteasome dependence (Figures 4D, S5A, and 5B). Immunofluorescence further confirmed PD‐L1 degradation via the proteasome pathway (Figures 4E and S5C–E). As proteasomal PD‐L1 degradation occurs predominantly at the protein level, we performed proteomic analysis on COR‐treated cells. The proteasome pathway ranked third among the affected pathways (Figure 4F). Additionally, endogenous immunoprecipitation confirmed that COR induced PD‐L1 ubiquitination (Figure 4G). These experimental results indicate that the degradation of PD‐L1 by COR is mediated through the ubiquitin–proteasome pathway.

Accumulating evidence indicates that PD‐L1 expression is critically regulated by ubiquitin‐mediated proteasomal degradation, suggesting that targeted modulation of PD‐L1 polyubiquitination could enhance the efficacy of immune checkpoint therapies [22]. Protein ubiquitination is a tightly controlled process mediated by coordinated interactions among E2 ubiquitin‐conjugating enzymes, E3 ubiquitin ligases, and deubiquitinating enzymes [23, 24]. Initially, ubiquitin is linked to the protein by the catalysis of E3 ubiquitin ligases, followed by the labeling of a ubiquitin chain onto the target protein, which then directs them toward degradation pathways. Among known E3 ligases, such as HRD1 [25], BTRC, STUB1 [26], MARCH8 [27], and SPOP [28], it has been reported that they can ubiquitinate PD‐L1 and promote its degradation. In RKO cells, COR was not able to degrade PD‐L1 only when HRD1 was knocked down by siRNA. These findings indicate that HRD1 may be a potential target of COR in the degradation of PD‐L1 (Figures 4H,I and S6A–H). Furthermore, IP experiments also revealed the interaction between HRD1 and PD‐L1 (Figure 4J,4K).

### 2.5 COR Targets the E3 Ligase HRD1 for Ubiquitination to Degrade PD‐L1

**FIGURE 5:**
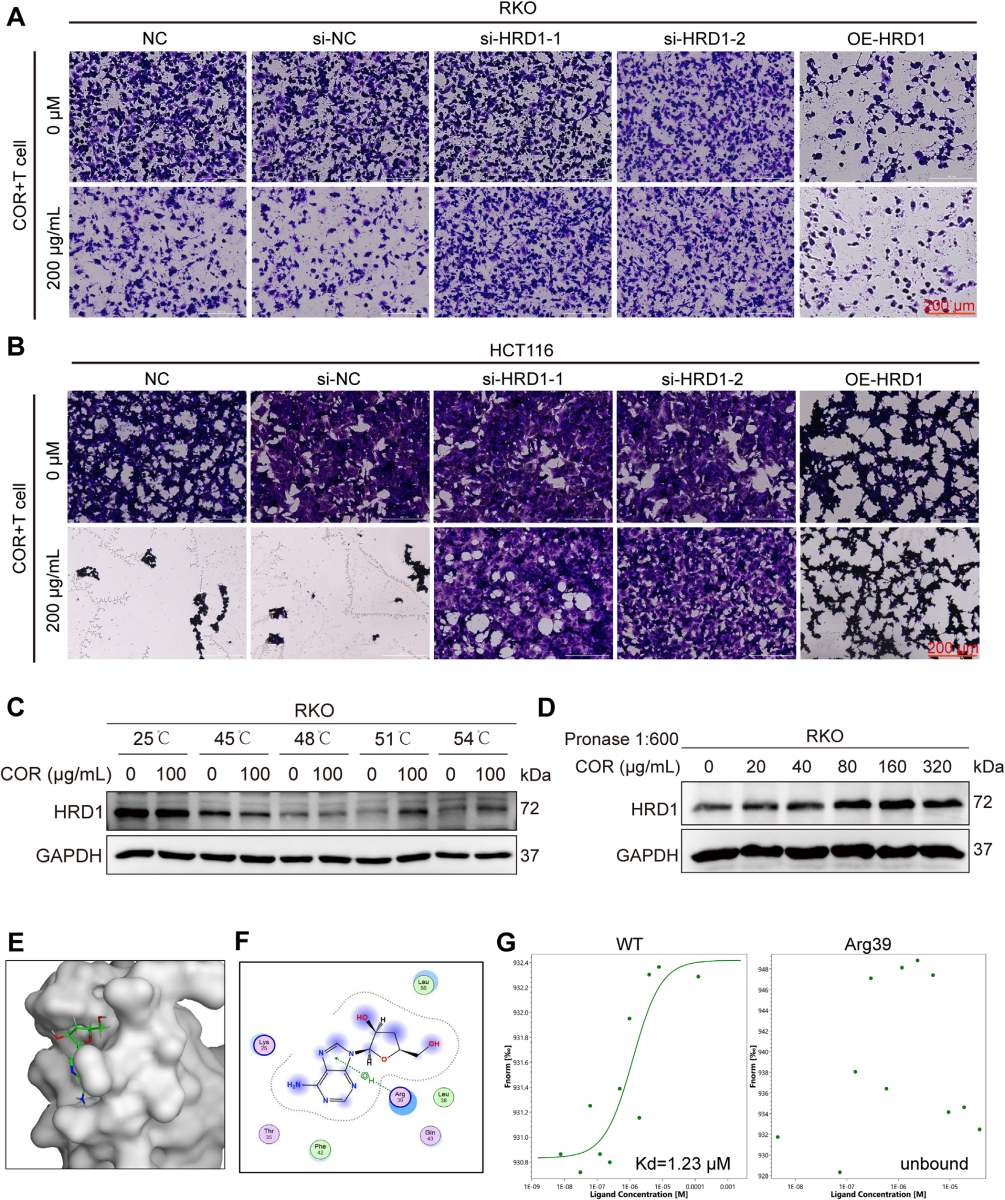
COR promotes in vitro T cell killing by targeting HRD1 ubiquitination to degrade PD‐L1. (A and B) Cell viability was measured via crystal violet staining following 24 h of coculture of RKO or HCT116 cells with Jurkat cells treated with/without COR. (C) Determination of the thermal stability of the interaction between COR and HRD1 in the temperature range of 25–54°C via the CETSA method. (D) Under the condition of a chain enzyme‐to‐protein ratio of 1:600, the stability of COR binding to HRD1 was detected. (E and F) Molecular docking of COR with HRD1. (G) Wild‐type HRD1 binds to COR, and COR binds to HRD1 mutated at Arg39.

To further confirm that COR enhances antitumor immunity by targeting HRD1 to degrade PD‐L1, we first used siHRD1 to knock down HRD1 in RKO and HCT116 cells. We found that siHRD1 treatment abrogated the COR‐mediated increase in T cell cytotoxicity in CRC cells. Moreover, overexpressing HRD1 in RKO or HCT116 cells did not augment the ability of COR to promote T cell‐mediated tumor cell killing (Figures 5A,5B and S7A,B). These results indicate that COR affects T cell cytotoxicity by targeting HRD1. To investigate potential direct binding between COR and HRD1, we initially conducted a cell thermal shift assay (CETSA) coupled with stringent wash conditions. Thermal stability profiling revealed COR‐induced stabilization of HRD1, with significant effects observed at temperatures exceeding 51°C (Figures 5C and S7C). Furthermore, concentration‐dependent accumulation of HRD1 was detected under optimized streptavidin–lysate binding conditions (1:600 ratio), demonstrating compound–protein interaction (Figures 5D and S7D). To identify the particular amino acid residues engaged in the interaction between COR and HRD1, molecular docking simulations were executed via MOE software. A favorable interaction was observed between COR and the Arg39 residue (Figure 5E,F). To precisely map the COR–HRD1 interaction domain, we generated GFP‐fused expression constructs encoding either wild‐type HRD1 or an Arg39 point mutant. Following transient transfection in 293T cells, we quantitatively assessed the kinetics of COR binding to GFP‐tagged HRD1 variants via microscale thermophoresis (MST). We subsequently examined the ability of the Arg39 mutant to bind COR and found that once Arg39 was mutated to alanine, the binding between COR and HRD1 disappeared (Figure 5G). In conclusion, our findings establish that the antitumor effects of COR are mediated through specific targeting and degradation of PD‐L1 via HRD1, with a critical interaction at the Arg39 residue, thereby enhancing T cell cytotoxicity against CRC cells.

### 2.6 COR is Comparable to either Anti‐CTLA4 Alone or Anti‐PD‐1 Alone for Antitumor Immunity

Clinical evidence has demonstrated that combining anti‐PD‐1 or anti‐CTLA‐4 immunotherapies with conventional treatments enhances therapeutic efficacy and prolongs patient survival across multiple cancer types [29]. COR functions as a PD‐1/PD‐L1 axis inhibitor through its ability to mimic anti‐PD‐1 activity. Based on this mechanism, we investigated potential synergistic interactions between COR and anti‐CTLA‐4 therapy.

**FIGURE 6:**
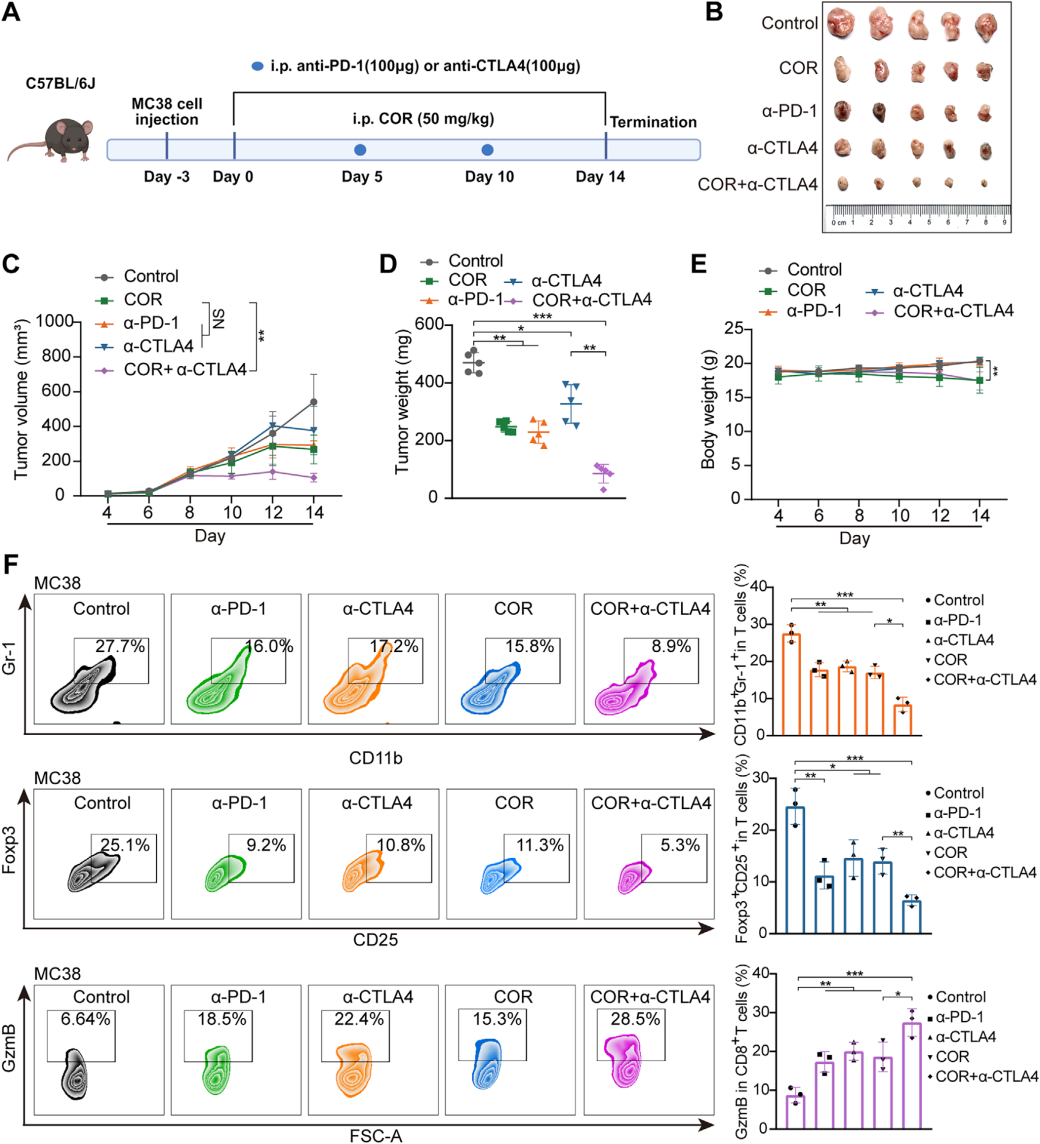
The combination of COR and anti‐CTLA4 has synergistic effects on antitumor therapy. (A) C57BL/6J (female) mouse animal experiment flowchart. (B) Therapeutic efficacy evaluation showing final tumor volumes following treatment with (i) an anti‐PD‐1 monoclonal antibody, (ii) an anti‐CTLA‐4 antibody, (iii) COR monotherapy (50 mg/kg), or (iv) COR (50 mg/kg) plus anti‐CTLA‐4 combination therapy. (C) Tumor growth patterns in C57BL/6J mice across different treatment groups. (D) Tumor weight data for C57BL/6J mice following various treatment regimens. (E) Body weight monitoring of C57BL/6J mice under different treatment conditions. (F) Flow cytometry was used to measure the levels of CD4^+^CD25^+^FoxP3^+^, CD11b^+^Gr‐1^+^, and CD3^+^CD8^+^ TILs in MC38 tumor tissues subjected to different treatments. The data are presented as the mean ± SEM, with *p < 0.05, **p < 0.01, and ***p < 0.001.

A subcutaneous CRC model was established by injecting MC38 cells into female C57BL/6J mice (Figure 6A). These mice were then administered COR, anti‐CTLA‐4, anti‐PD‐1, or a combination of COR and anti‐CTLA‐4. These findings demonstrated that single‐agent COR treatment exhibited efficacy comparable to that of anti‐PD‐1 treatment. Moreover, combination therapy was significantly more potent at suppressing tumor growth than either COR or anti‐CTLA‐4 when used alone (Figure 6B–E). Flow cytometry examination of the tumor tissues from the mice revealed that the group receiving the combination therapy presented the smallest quantities of myeloid‐derived regulatory T cells (Treg cells; CD4^+^CD25^+^Foxp3^+^) and suppressor cells (MDSCs; CD11b^+^Gr‐1^+^) but the highest concentration of granzyme B (GzmB) (Figure 6F). These findings suggest that the combined treatment significantly diminished the number of immunosuppressive cell types and augmented the cytotoxic activity within the tumor microenvironment. Immunohistochemical results also showed that the level of PD‐L1 in tumor tissue was significantly reduced after COR treatment, further confirming the immunomodulatory effect of COR. In addition, combination therapy with COR and anti‐CTLA4 not only increased the infiltration of T cells but also promoted the apoptosis of tumor cells (Figure S8A). H&E staining of mouse viscera confirmed drug safety across all treatment groups (Figure S8B). These results suggest that COR combined with anti‐CTLA4 therapy can synergistically suppress CRC cell proliferation and enhance antitumor efficacy.

### 2.7 Association of HRD1 with the Clinical Management of Colon Cancer

**FIGURE 7:**
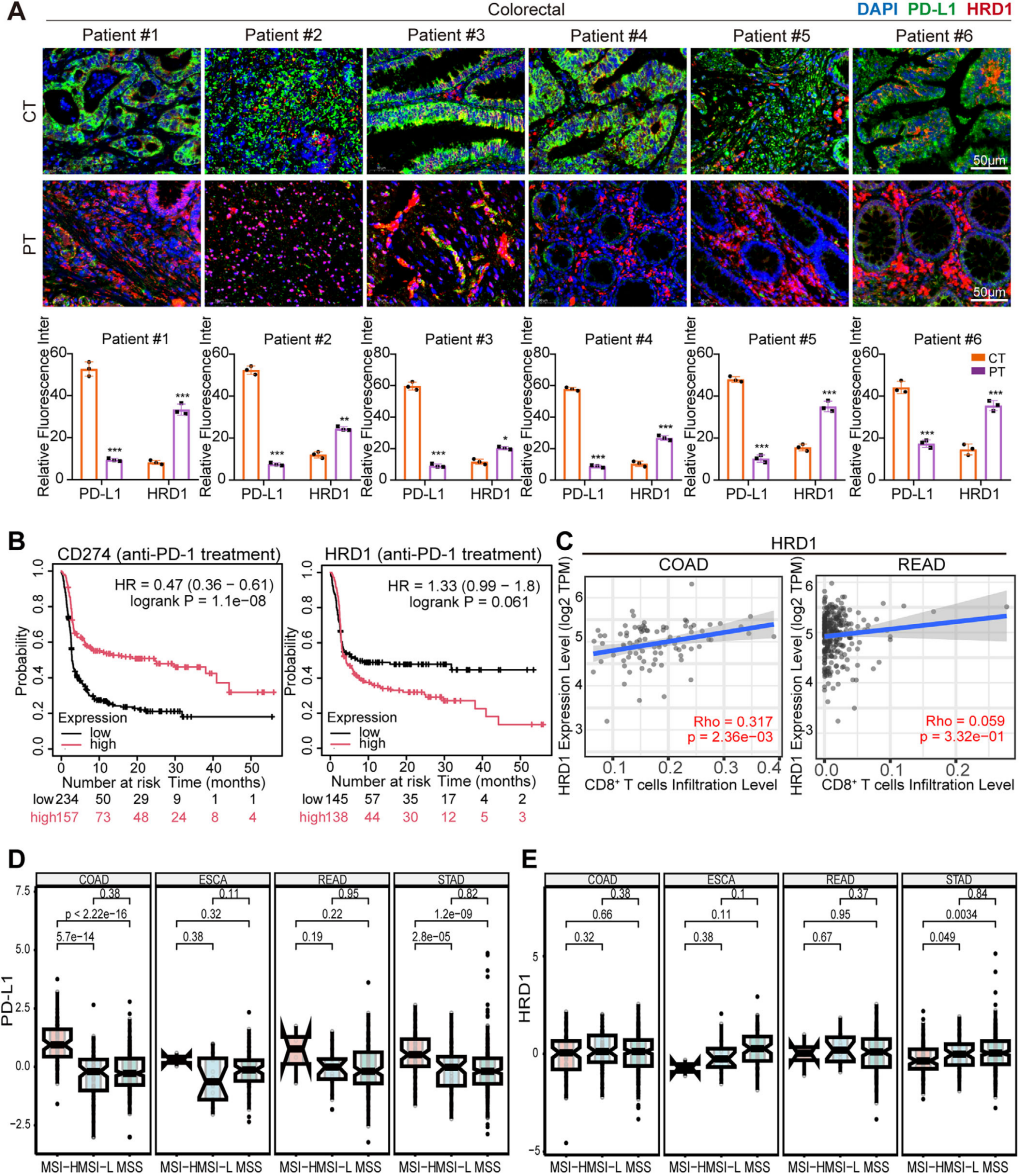
The clinical importance of HRD1 in therapeutic approaches for colon cancer patients. (A) Fluorescently double‐stained IHC was used to examine the levels of PD‐L1 and HRD1 expression in cancerous (CT) and paraneoplastic (PT) tissues from patients with CRC. The data are presented as the mean ± SEM, with *p < 0.05, **p < 0.01, and ***p < 0.001. (B) Comparison of survival outcomes among colorectal cancer (CRC) patients stratified on the basis of CD274 expression and HRD1 expression via two‐sided log rank analysis. (C) TIMER 2.0 scatter plots were used to show the correlation between HRD1 expression and CD8^+^ T cell infiltration in COAD or READ patients. (D) PD‐L1 expression in patients with different MSs (microsatellite instability). (E) Evaluation of HRD1 expression across various molecular isoforms (microsatellite instability).

To explore HRD1 and PD‐L1 expression in human tumor tissues, we assessed their protein levels in paired adjacent and tumor tissues from six colon cancer patients. Figure 7A reveals increased HRD1 expression in surrounding tissues and increased PD‐L1 expression in tumor tissue. The Kaplan–Meier plotter data indicate that in CRC patients, the efficacy of anti‐PD‐1 immunotherapy was proportional to the expression level of PD‐L1 and inversely proportional to the expression of HRD1. Patients with low HRD1 and high PD‐L1 expression were more likely to experience prolonged survival (Figure 7B). TIMER 2.0 was used to evaluate the interaction between immune cell infiltration and the levels of HRD1 and PD‐L1 in colorectal adenocarcinoma (COAD) and rectal adenocarcinoma (READ) patients. As shown in Figures 7C and S9A, CD8^+^ T cell infiltration was negatively correlated with PD‐L1 expression but positively correlated with HRD1 expression. These results suggest that tumors with elevated PD‐L1 expression and reduced HRD1 expression might inhibit T cell activation within the tumor immune microenvironment. Furthermore, HRD1 was positively associated with pivotal components of the cancer immune cycle. These components include tumor immune activation stages, antigen release, immune cell infiltration (such as CD4^+^ T cells, macrophages, dendritic cells, and NK cells), and T cell‐mediated recognition of cancer cells in COAD and READ (Figures S9B,C and S10A–D). In colon cancer patients, the high‐frequency microsatellite instability (MSI‐H) molecular subtype is linked to a more favorable immune response [30]. As shown in Figure 7D,E, PD‐L1 levels were elevated in the MSI‐H molecular subtype, COAD, esophageal cancer, and READ samples and were inversely related to HRD1 expression. However, this inverse relationship was not observed in gastric adenocarcinoma or endometrioid adenocarcinoma. In summary, our results suggest that HRD1 may have an inhibitory effect on the expression of PD‐L1 in tumor tissues.

## 3 Discussion

Therapies targeting PD‐1/PD‐L1 immune checkpoints enhance T cell‐mediated antitumor responses. They achieve this by blocking PD‐1/PD‐L1 binding and play a crucial role in cancer immunotherapy. Similarly, small‐molecule agents that induce PD‐L1 degradation offer advantages analogous to those of PD‐1 monoclonal antibodies [31, 32]. In addition, small‐molecule drugs efficiently block the PD‐1/PD‐L1 signaling pathway by regulating PD‐L1 posttranslational modifications and destabilizing and degrading PD‐L1 to disrupt the PD‐1/PD‐L1 axis. Compared with monoclonal antibodies, small‐molecule drugs offer significant benefits, including better oral bioavailability, deeper tissue penetration, controllable half‐life, cost effectiveness, diverse mechanisms of action, and greater flexibility in combination therapies [33, 34, 35]. Here, we comprehensively analyzed the effect of CME on PD‐L1 expression in human CRC cells. Through activity tracing, we found that the active component in CME that reduces PD‐L1 activity is COR. Next, via Western blotting, flow cytometry, and immunofluorescence experiments, we demonstrated that COR significantly reduced total PD‐L1 and membrane‐associated PD‐L1 protein levels in RKO and HCT116 cells in a concentration‐ and time‐dependent manner. Mechanistic studies revealed that COR facilitates PD‐L1 degradation by directly targeting HRD1. This process blocks the PD‐1/PD‐L1 immune checkpoint pathway by preventing PD‐1 on T cells from interacting with tumor‐associated PD‐L1. In turn, this blockage results in two positive outcomes: (1) reinvigorating the intratumoral immune microenvironment and (2) boosting systemic antitumor immunity. Furthermore, the results from animal experiments also support that COR enhances T cell‐mediated killing by degrading PD‐L1 in tumor cells, thereby exerting its antitumor effects.

Previous studies have shown that many natural small‐molecule compounds exert antitumor effects via PD‐L1‐related pathways. For example, resveratrol can activate the E3 ubiquitin ligase β‐TrCP in triple‐negative breast cancer cells, thereby decreasing PD‐L1 protein levels in these cells [36]; lycopene can also directly induce the expression of β‐TrCP in cancer cells, promoting the degradation of PD‐L1 [37]. Furthermore, COR has been shown to reshape the tumor microenvironment of CRC by downregulating PD‐L1 expression, but the specific mechanism has not yet been fully elucidated [38]. Research has revealed that COR downregulates PD‐L1 transcription via the NOD‐like receptor/NF‐κB/STAT1 pathway, which inhibits glioma growth. However, its specific targets remain unclear [39]. In contrast to previous studies, our results indicate that COR promotes PD‐L1 degradation through the ubiquitin‒proteasome pathway, which was confirmed by the reversal of COR‐induced PD‐L1 degradation by the proteasome inhibitor MG132. This discovery is highly important because it highlights the potential of targeting ubiquitin ligases for the degradation of PD‐L1 as a therapeutic strategy. Through molecular docking and cellular thermal shift assays, we found that COR may specifically target the Arg39 site of HRD1, an E3 ubiquitin ligase. This interaction promotes PD‐L1 ubiquitination and degradation, enhancing antitumor immunity. These findings lay the theoretical groundwork for developing COR as a PD‐L1 inhibitor and enhancing the understanding of its pharmacological mechanisms.

The management of solid tumors faces substantial obstacles due to the dual mechanisms of immune escape and immunosuppression within the tumor microenvironment. Disrupting the PD‐L1/PD‐1 axis has emerged as a novel therapeutic paradigm to restore T cell‐driven tumor elimination. Preclinical evidence reveals COR's dual immunomodulatory effects: it simultaneously suppresses PD‐L1 expression in neoplastic cells and facilitates CD8^+^ T lymphocyte recruitment into tumor sites, synergistically amplifying host defenses against malignancies. In addition to these direct actions, COR exerts secondary immunoenhancement effects by limiting the expansion of MDSCs and Treg cells, thereby alleviating the microenvironmental suppression of cytotoxic lymphocytes. Notably, COR does not induce significant cytotoxicity within its effective concentration range, demonstrating its potential as a safe and effective therapeutic candidate. Immune checkpoint blockade strategies, which target the PD‐1/PD‐L1 and CTLA4/B7‐1/2 immunosuppressive pathways, have shown significant therapeutic effects in clinical settings. Given that COR can block the PD‐L1/PD‐1 pathway, we hypothesize that the combined use of COR and anti‐CTLA‐4 antibodies may further enhance the tumor immune response [40, 41, 42, 43]. In the MC38 mouse model, compared with monotherapy with COR or anti‐CTLA4 antibodies alone, the combination treatment significantly increased the infiltration of CD8^+^ T cells into the tumor and led to a substantial reduction in tumor volume, accompanied by severe tumor necrosis. Additionally, combination therapy decreased the number of immunosuppressive cells, such as MDSCs and Treg cells, and increased the levels of GzmB, a sign of cytotoxic T cell activation [44, 45, 46]. Our findings indicate that COR combined with anti‐CTLA4 antibodies holds promise for antitumor immunotherapy. Our clinical analyses revealed a significant inverse correlation between HRD1 and PD‐L1 expression levels in CRC samples, with elevated HRD1 expression consistently associated with reduced PD‐L1 protein abundance. This regulatory relationship, coupled with the ability of COR to suppress PD‐L1 through targeting HRD1, strongly suggests that HRD1‐mediated regulation of PD‐L1 is involved in CRC pathogenesis.

In summary, our study provides evidence that COR, as an active component of CME, downregulates PD‐L1 expression in CRC cells through targeting the HRD1‐mediated ubiquitin‒proteasome pathway, thereby enhancing T‐cell‐mediated antitumor immunity. The observed synergistic effect of COR in combination with anti‐CTLA4 therapy highlights the potential of this approach to increase the efficacy of CRC immunotherapy. This combined therapy has the potential to improve patient treatment outcomes, opening new uses for COR and offering better clinical prospects for CRC patients.

## 4 Materials and Methods

### 4.1 Western Blotting and Immunoprecipitation

The cells were plated at a density of 4 × 10⁵ cells/well in six‐well plates, treated with compounds for 24 h, and harvested. Total protein was extracted via RIPA buffer (Beyotime, China) containing 1% protease inhibitor, quantified via a BCA assay (Beyotime), and resolved by SDS‐PAGE. Following electrophoretic transfer to PVDF membranes, the membranes were blocked for 1 h with 5% skim milk and subsequently incubated with primary antibodies overnight at 4°C. After washing, secondary antibody incubation was performed for 1 h at room temperature. The protein bands were finally visualized via a Bio‐Rad imaging system.

Cellular extracts were generated via the use of IP lysis buffer (Beyotime) containing a 1% protease inhibitor cocktail. Following overnight incubation at 4°C with target‐specific antibodies, the immune complexes were subjected to five wash cycles prior to Western blot analysis. Antibody dilutions (Table S1) were performed according to the manufacturer's specifications.

### 4.2 RT‐PCR Analysis

Cellular RNA was isolated via RNAiso Plus reagent (Takara Bio), followed by cDNA synthesis via the PrimeScript RT Kit (Takara Bio) according to the manufacturer's thermal cycling protocol. Quantitative PCR amplification was performed on a LightCycler 96 system (Roche Diagnostics) with β‐actin as the endogenous normalization control. The primer pair sequences are listed in Table S2.

### 4.3 Transfection

Transfection was performed when the cells reached over 50% confluence via Lipofectamine 2000 (Invitrogen, Carlsbad, CA) following the manufacturer's instructions. The procedures included (1) NC siRNA or specific siRNA (GenePharma, Shanghai, China; sequences are listed in Table S3) and (2) the pcDNA3.1‐Ub plasmid (GenePharma). After 8 h of transfection, the medium was changed to complete medium supplemented with serum and bispecific antibodies. The cells were then cultured for 24 h before being subjected to drug treatment for another 24 h. All other experimental settings adhered to standard siRNA transfection protocols.

### 4.4 T‐Cell‐Mediated Tumor Cell Killing Assay

RKO and HCT116 colorectal carcinoma cells were plated in 12‐well plates (2.5 × 10⁵ cells/well) and maintained until full adhesion and >50% monolayer formation was achieved. At this confluency stage, the experimental compounds were applied for 24 h. Activated Jurkat T cells (with an effector‐to‐target ratio of 1:9) were subsequently introduced alongside a T cell activation cocktail (1 µg/mL phytohemagglutinin + 50 ng/mL phorbol myristate acetate). Following 48 h of coculture, residual adherent tumor cell viability was assessed through crystal violet retention assays, with stained monolayers documented via a Cytation 5 multimodal reader (BioTek).

### 4.5 Cell Thermal Displacement Measurement

Following the lysis of RKO cells in RIPA buffer, the homogenate was divided into equal aliquots. The experimental samples were supplemented with 100 µg/mL COR, whereas the vehicle controls were adjusted with PBS to maintain equivalent volumes. After 10 min of end‐over‐end mixing at ambient temperature, the reaction mixtures were dispensed into 100 µL PCR tubes (50 µL/tube) and subjected to temperature gradient incubation (3 min at specified thermal conditions). Postequilibration processing included rapid cooling on ice; high‐speed centrifugation (12,000×*g*, 10 min, 4°C); and supernatant collection for buffer formulation with 5× SDS loading buffer (4:1). Denaturation at 95°C (10 min) preceded SDS‐PAGE resolution and standard IB protocols.

### 4.6 Microcalorimetry

Recombinant GFP‐tagged target proteins were expressed in 293T cells through transfection with a GFP‐HRD1 overexpression plasmid. Following 48 h of transfection, cellular proteins were extracted via IP lysis buffer supplemented with protease inhibitors. The resulting lysates were clarified by centrifugation at 12,000×*g* for 15 min at 4°C prior to subsequent analyses. Freshly prepared gradient concentrations of drugs were mixed with the cell lysate and measured via MST equipment (NanoTemper, Germany), Monolith TM NT.115.

### 4.7 Clinical Tissue Samples

All patients received treatment at Longhua Hospital, Shanghai University of Traditional Chinese Medicine (Shanghai, China). Written informed consent was obtained from patients and/or their parents prior to sample collection. The Ethics Committee of Longhua Hospital, Shanghai University of Traditional Chinese Medicine, approved the study protocol (2025LCSY045).

### 4.8 Data Availability and Analysis

GraphPad Prism 9.0.1 was used for statistical analysis and data visualization. Student's *t*‐test was employed for pairwise comparisons, and one‐way ANOVA was used for multigroup comparisons. Two‐way ANOVA and repeated‐measures ANOVA were used to analyze tumor growth kinetics. ImageJ was used for quantitative data processing.

## Author Contributions

Sanhong Liu, Weidong Zhang, and Qun Wang: conceptualization, original draft, methodology, review and editing, funding acquisition, and supervision. Xiangxin Geng, Minchen Cai, and Hongmei Hu carried out the experiments, generated the figures, and wrote the paper. Mengting Xu, Qing Zhang, Hanchen Xu, Dianping Yu, Hongwei Zhang, Mengmeng Guo, Hanchi Xu, Linyang Li, and Shize Xie participated in part of the experiments. All authors have read and approved the final manuscript.

## Conflicts of Interest

The authors declare no conflicts of interest.

## Ethics Statement

All animal experiments were approved by the Ethics Committee of the Department of Laboratory Animal Science at Shanghai University of Traditional Chinese Medicine (SHUTCM). Approval No: PZSHUTCM2302140003; PZSHUTCM2211280003.

## Supporting information




**Figure S1**: CME can reduce the expression level of PD‐L1 in colorectal cancer. (A and B) Flow cytometry results showing that CME reduces PD‐L1 expression levels in RKO cells. (C and D) Flow cytometry analysis revealed that CME reduces PD‐L1 expression in HCT116 cells. (E–H) After RKO and HCT116 cells were treated with different concentrations of CME for 24 h, the expression of PD‐L1 on the cell membrane surface was detected via immunofluorescence. Red represents PD‐L1, blue represents the nucleus, and the scale bar represents 200 µm. Immunofluorescence analysis revealed that CME reduces PD‐L1 expression in RKO and HCT116 cells. Statistical differences were determined via Student's *t*‐test. *p < 0.05; **p < 0.01; ***p < 0.001; NS, not significant.
**Figure S2**: The impact of CME on cytotoxicity. (A–C) Detection of CME toxicity in RKO cells, HCT116 cells, and NCM460 cells via a CCK‐8 assay. (D) RKO and HCT116 cells were treated with CME for 24 h. The effects of the drugs on the cells were detected via an EdU kit. (Scale bar = 200 µm). (E and F) Analysis of the ability of CME to promote the killing of RKO cells and HCT116 cells by Jurkat cells. Statistical differences were determined via Student's *t*‐test. *p < 0.05; **p < 0.01; ***p < 0.001; NS, not significant.
**Figure S3**: Isolation and confirmation of PD‐L1‐reducing active ingredients in CME. (A) The effects of different concentrations of the CME components C1 and C2 on the surface PD‐L1 of the RKO cell membrane were detected via flow cytometry. (B and C) Detection of the effects of the C2 components W1‐W9 on PD‐L1 expression on the surface of the RKO cell membrane via flow cytometry. (D) The chemical structure of cordycepin. (E and F) An immunofluorescence assay was used to detect the effect of COR on PD‐L1 expression on the membrane surface of RKO and HCT116 cells. (G and I) A CCK‐8 experiment was conducted to detect the cytotoxic effects of COR on RKO cells, HCT116 cells, and NCM460 cells at effective concentrations. Statistical differences were determined via Student's *t*‐test. *p < 0.05; **p < 0.01; ***p < 0.001; NS, not significant.
**Figure S4**: The killing effect of COR on cancer cells in vitro and in vivo. (A and B) Analysis of COR‐mediated promotion of the killing ability of Jurkat cells against RKO cells and HCT116 cells. (C) H&E staining of major organs in C57BL/6J mice in the control group or COR. (D) Results of IHC staining for CD8, cleaved cysteine protease‐3, FOXP3, PD‐L1, and Ki‐67. (E) Flowchart of the animal experiments involving immunodeficient mice (female). Statistical differences were determined via Student's *t*‐test. NS, not significant, ^#^ *p *< 0.05 compared with the RKO or HCT116 DMSO group; *p < 0.05; **p < 0.01; ***p < 0.001 compared with the RKO + Jurkat cell or HCT116 + Jurkat cell group.
**Figure S5**: The ubiquitin–proteasome inhibitor MG132 can reverse the degradation of PD‐L1 by COR. (A and B) Effects of the combination of COR, MG132, and CQ inhibitors on PD‐L1 levels in HCT116 cells. (C–E) An immunofluorescence assay was used to observe the changes in PD‐L1 protein expression on the surface of HCT116 cell membranes when COR was used in combination with MG132 or CQ. Statistical differences were determined by Student's *t*‐test. *p < 0.05; **p < 0.01; ***p < 0.001; NS, not significant.
**Figure S6**: The effect of COR and E3 ligase co treatment on PD‐L1 expression level. (A–H) RKO cells were treated with siRNAs targeting BTRC, SPOP, STUB, MARCH8, or control siRNA, followed by treatment with COR, and changes in PD‐L1 expression were detected via protein immunoblotting. Quantitative RT‐PCR was used to detect the knockdown efficiency of the siRNAs, and statistical analysis of the immunoblot results was performed. Statistical significance is denoted as *p < 0.05, **p < 0.01, and ***p < 0.001.
**Figure S7**: The interaction relationship between COR and HRD1. (A and B) Statistical chart of T cell killing under the action of COR and HRD1. (C) Statistical chart of CETSA experiment. (D) Statistical chart of streptavidin experiment. The statistical significance is expressed as *p < 0.05, **p < 0.01, and ***p < 0.001.
**Figure S8**: The combined application of COR and anti‐CTLA4 has an impact on the immune microenvironment. (A) Results of IHC staining for CD8, cleaved cysteine protease‐3, FOXP3, PD‐L1, and Ki‐67. (Scale bar = 100 µm). (B) Hematoxylin and eosin staining of major organs in C57BL/6J mice treated with control or COR. (Scale bar = 200 µm). The data shown are mean ± standard error (SEM) *p < 0.05, **p < 0.01, and ***p < 0.001.
**Figure S9**: Clinical relevance of HRD1 in the treatment of patients with colon cancer. (A) Visualization of the correlation between PD‐L1 expression and infiltrating CD8^+^ T cells in COAD or READ patients via scatter plots generated with TIMER 2.0. (B and C) Correlations between the levels of different immune cell types and PD‐L1 expression in patients with COAD.
Figure S10 The clinical correlation between HRD1 and cancer treatment. (A and B) Correlations between the levels of different immune cell types and PD‐L1 expression in patients with COAD. (C and D) Correlations between PD‐L1 expression and steps of the cancer immune cycle in patients with COAD.
**Table S1**: Antibody.
**Table S2**: Primers for qRT‒PCR.
**Table S3**: siRNA sequences used for knocking down the indicated proteins.

## Data Availability

All data are available from the corresponding authors upon request.
