# Supplementary material for: Cordycepin Targets HRD1 to Promote Cancer Cell PD‐L1 Ubiquitin–Proteasome Degradation and Increase Antitumor Immunity
Source: MedComm (2020). 2025 Oct 17;6(11):e70430. doi: 10.1002/mco2.70430 (PMC12531455; doi:10.1002/mco2.70430)
Supplement: Supplementary file 1 — Figure S1: CME can reduce the expression level of PD‐L1 in colorectal cancer. (A and B) Flow cytometry results showing that CME reduces PD‐L1 expression levels in RKO cells. (C and D) Flow cytometry analysis revealed that CME reduces PD‐L1 expression in HCT116 cells. (E–H) After RKO and HCT116 cells were treated with different concentrations of CME for 24 h, the expression of PD‐L1 on the cell membrane surface was detected via immunofluorescence. Red represents PD‐L1, blue represents the nucleus, and the scale bar represents 200 µm. Immunofluorescence analysis revealed that CME reduces PD‐L1 expression in RKO and HCT116 cells. Statistical differences were determined via Student's t‐test. *p < 0.05; **p < 0.01; ***p < 0.001; NS, not significant. Figure S2: The impact of CME on cytotoxicity. (A–C) Detection of CME toxicity in RKO cells, HCT116 cells, and NCM460 cells via a CCK‐8 assay. (D) RKO and HCT116 cells were treated with CME for 24 h. The effects of the drugs on the cells were detected via an EdU kit. (Scale bar = 200 µm). (E and F) Analysis of the ability of CME to promote the killing of RKO cells and HCT116 cells by Jurkat cells. Statistical differences were determined via Student's t‐test. *p < 0.05; **p < 0.01; ***p < 0.001; NS, not significant. Figure S3: Isolation and confirmation of PD‐L1‐reducing active ingredients in CME. (A) The effects of different concentrations of the CME components C1 and C2 on the surface PD‐L1 of the RKO cell membrane were detected via flow cytometry. (B and C) Detection of the effects of the C2 components W1‐W9 on PD‐L1 expression on the surface of the RKO cell membrane via flow cytometry. (D) The chemical structure of cordycepin. (E and F) An immunofluorescence assay was used to detect the effect of COR on PD‐L1 expression on the membrane surface of RKO and HCT116 cells. (G and I) A CCK‐8 experiment was conducted to detect the cytotoxic effects of COR on RKO cells, HCT116 cells, and NCM460 cells at effective concentrations. St [file MCO2-6-e70430-s001.docx]

**Cordycepin targets HRD1 to promote cancer cell PD-L1 ubiquitin proteasome degradation and increase antitumor immunity**

Xiangxin Geng^1#^, Minchen Cai^1#^, Hongmei Hu^1#^, Mengting Xu^1^, Qing Zhang^1^, Hanchen Xu^2^, Dianping Yu^1^, Hongwei Zhang^1^, Hanchi Xu^1^, Linyang Li^1^, Mengmeng Guo^1^, Shize Xie^3^, Qun Wang^1*^, Weidong Zhang^3,4,5*^, Sanhong Liu^1*^

^1^ State Key Laboratory of Discovery and Utilization of Functional Components in Traditional Chinese Medicine, Shanghai Frontiers Science Center of TCM Chemical Biology, Institute of Interdisciplinary Integrative Medicine Research, Shanghai University of Traditional Chinese Medicine, Shanghai, China

^2^ Institute of Digestive Diseases, Longhua Hospital, Shanghai University of Traditional Chinese Medicine, Shanghai, China

^3^ Department of Phytochemistry, School of Pharmacy, Second Military Medical University, Shanghai, China

^4^ Institute of Medicinal Plant Development, Chinese Academy of Medical Sciences and Peking Union Medical College, Beijing, China

^5^ The Research Center for Traditional Chinese Medicine, Shanghai Institute of Infectious Diseases and Biosafety, Institute of Interdisciplinary Integrative Medicine Research, Shanghai University of Traditional Chinese Medicine, Shanghai, China

^#^ These authors contributed equally to this work.

*Corresponding authors: Sanhong Liu, liush@shutcm.edu.cn; Weidong Zhang, wdzhangy@hotmail.com; Qun Wang, Qunwang0523@163.com

**1 MATERIALS AND METHODS**

**1.1 Flow cytometry and immunofluorescence**

Post-treatment cell suspensions were labeled with an anti-PD-L1 monoclonal antibody (30 min, 4°C) following drug exposure. After three washes with PBS, the samples were reconstituted in 500 μL of light-protected PBS for flow cytometric quantification of surface PD-L1 expression.

For membrane-localized PD-L1 quantification, cells were plated in 12-well culture plates and treated with drugs at >50% confluency. Post-treatment protocols included paraformaldehyde fixation, 5% BSA blocking, overnight primary antibody incubation (12 hours, 4°C), and fluorescence microscopy via the Cytation 5 system (DAPI nuclear counterstain). Antibody specifications are provided in Supplementary Table S1.

**1.2 Cell viability and toxicity testing**

For cytotoxicity evaluation, 5 × 10³ cells/well were plated in 96-well plates and allowed to achieve 80% confluency prior to 24 h of exposure to a dose-response gradient of test compounds. After treatment, the medium was replaced with serum-free solution containing 10% CCK-8 reagent (Beyotime Institute of Biotechnology, China), followed by a 1-4 h incubation at 37°C. The optical density at 450 nm was determined via a Synergy H1 microplate reader (BioTek), with dose-response curves and IC50 values generated via four-parameter logistic regression.

Proliferative activity was assessed by seeding cells in 12-well plates (70% confluency at treatment initiation) for 24 h of drug exposure. Post-treatment processing included 2 h incubation with EdU working solution (Beyotime, China), methanol fixation, 0.5% Triton X-100 permeabilization, and Click-iT reaction cocktail staining following the manufacturer’s specifications. Nuclei were counterstained with Hoechst 33342, and proliferation indices were calculated from 5 fields/well via the ImageXpress Micro XLS system (Molecular Devices).

**1.3 Animal experiments**

All animal procedures were performed under the ethical guidelines of Shanghai University of Traditional Chinese Medicine (SHUTCM) and approved by its Institutional Animal Care and Use Committee. Female C57BL/6J mice and nude mice (6-8 weeks old) were acquired from Shanghai Jihui Laboratory Animal Co. Subcutaneous implantation of 1 × 10⁶ log-phase MC38 colorectal carcinoma cells were performed in the right flank of all the animals. Therapeutic interventions commenced upon achieving baseline tumor volumes of 50 mm³, which were calculated via the ellipsoid formula (L × W²/2). COR was formulated in a vehicle solution containing 10% dimethyl sulfoxide (DMSO), 60% phosphate-buffered saline (PBS), and 30% polyethylene glycol 300 (PEG300), with intraperitoneal delivery at 24-hour intervals. The mice were treated with either anti-CTLA-4 (100 μg) or anti-PD-1 (100 μg) monoclonal antibodies every 5 days. Tumor dimensions were measured twice weekly via digital calipers, with volumes calculated as (length × width²)/2. Upon study completion, excised tumors underwent comprehensive analysis, including (1) immunohistochemical evaluation of immune markers, (2) flow cytometric profiling of tumor-infiltrating lymphocytes, and (3) histological examination of major organs via hematoxylin-eosin (H&E) staining. For detailed information on the relevant antibodies, please refer to Supplementary Table S1.

**1.4 Isolation of tumor-infiltrating lymphocytes and analysis of T cells**

The tumor samples were subjected to enzymatic dissociation via the use of 1 mg/mL collagenase IV and 0.1 mg/mL DNase I (Yeasen Biotechnology) in a 37°C shaking water bath (2 h).Resultant single-cell suspensions were subjected to sequential washing (300 × g, 5 min) and 70 μm nylon mesh filtration and stained with fluorochrome-conjugated antibodies targeting GzmB, CD11b, CD25, Gr-1, CD8, CD3, and Foxp3 (4°C, 30 min). After the samples were washed with PBS (2% serum), they were analyzed on a Beckman Coulter flow cytometer. The data were processed via FlowJo, and the antibody details are provided in Supplementary Table S4.

**1.5 Proteomic analysis**

RKO cells were cultured in 10 cm culture dishes and incubated until the cell density reached 70%-80%. Afterward, the cells were treated with COR (100 µg/mL) or an equal volume of DMSO for 24 h. Each sample was subjected to an SDT lysis procedure. According to the requirements of the process, all the samples were digested with trypsin at 37 for 16-18 h. The remaining steps were followed by LC-MS/MS. The data is stored in the iProX database with the login number [PXD065804].


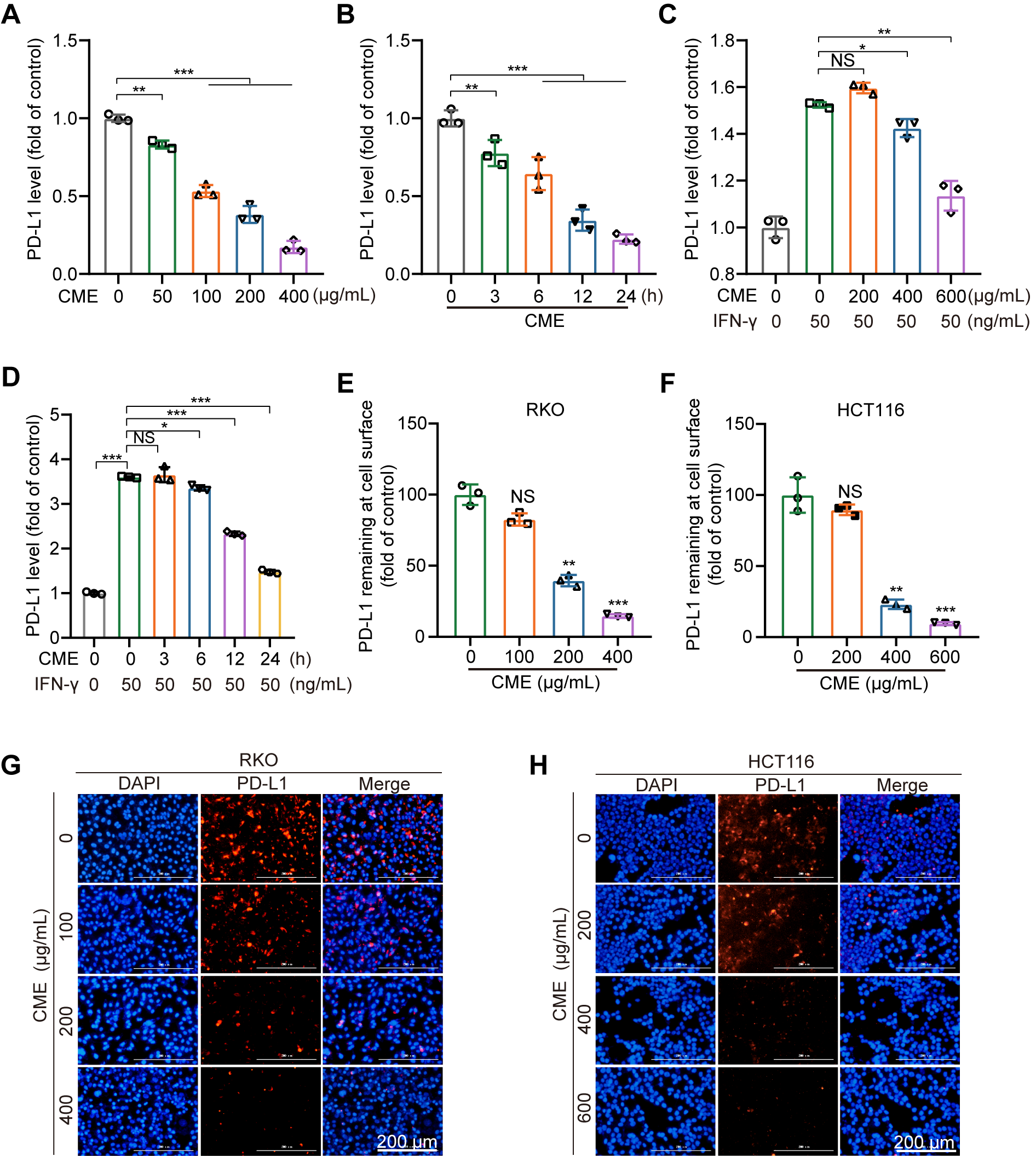


**Figure S****1.** **CME can reduce the expression level of PD-L1 in colorectal cancer. (A-B)** Flow cytometry results showing that CME reduces PD-L1 expression levels in RKO cells. **(C-D)** Flow cytometry analysis revealed that CME reduces PD-L1 expression in HCT116 cells. **(E-H)** After RKO and HCT116 cells were treated with different concentrations of CME for 24 h, the expression of PD-L1 on the cell membrane surface was detected via immunofluorescence. Red represents PD-L1, blue represents the nucleus, and the scale bar represents 200 μm. Immunofluorescence analysis revealed that CME reduces PD-L1 expression in RKO and HCT116 cells. Statistical differences were determined via Student’s t test. **p* < 0.05; ***p* < 0.01; ****p* < 0.001; NS, not significant.


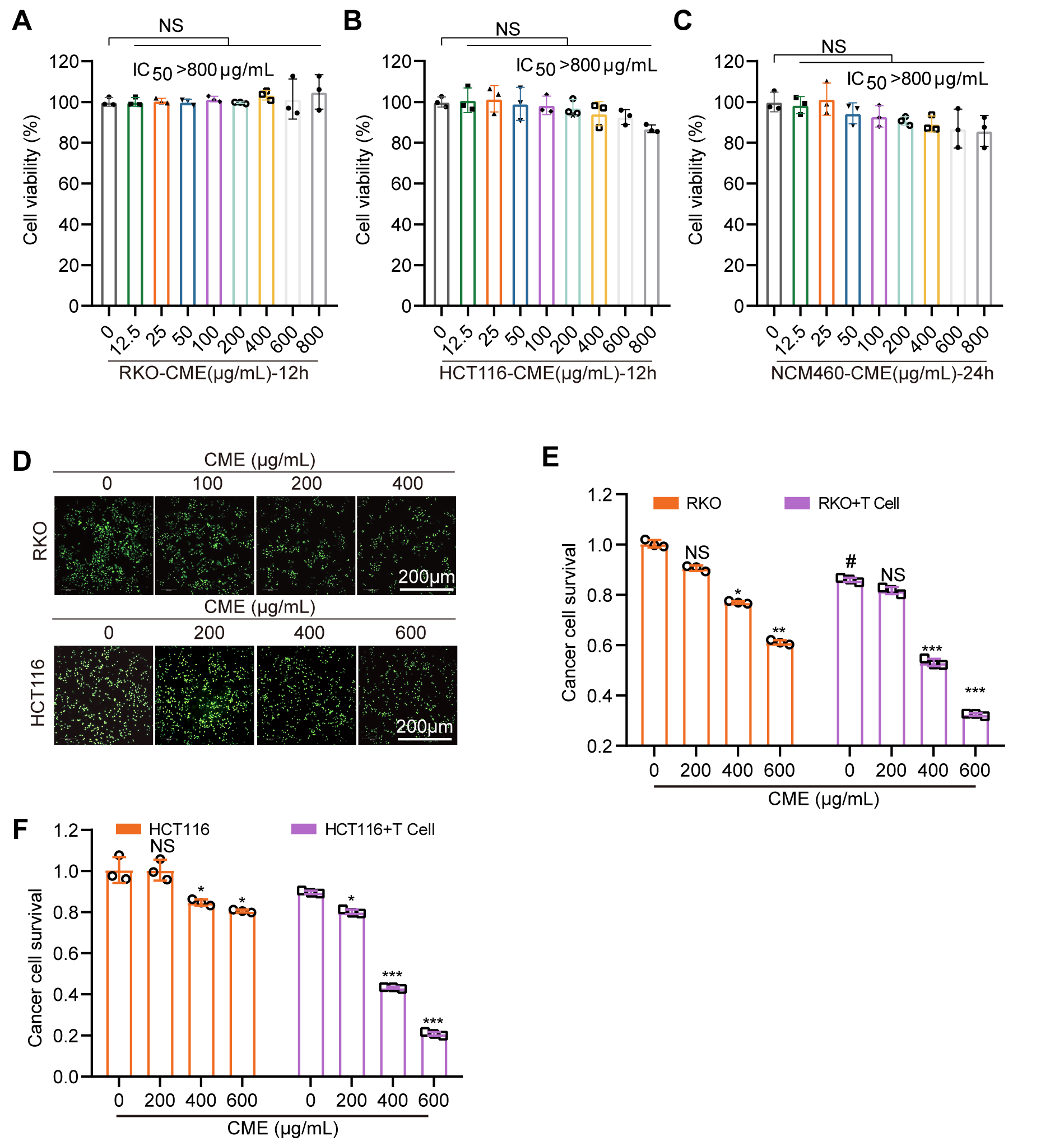


**Figure S2. The impact of CME on cytotoxicity. (A-C)** Detection of CME toxicity in RKO cells, HCT116 cells and NCM460 cells via a CCK-8 assay. **(D)** RKO and HCT116 cells were treated with CME for 24 h. The effects of the drugs on the cells were detected via an EdU kit. (Scale bar = 200 μm). **(E-F)** Analysis of the ability of CME to promote the killing of RKO cells and HCT116 cells by Jurkat cells. Statistical differences were determined via Student’s t test. **p* < 0.05; ***p* < 0.01; ****p* < 0.001; NS, not significant.


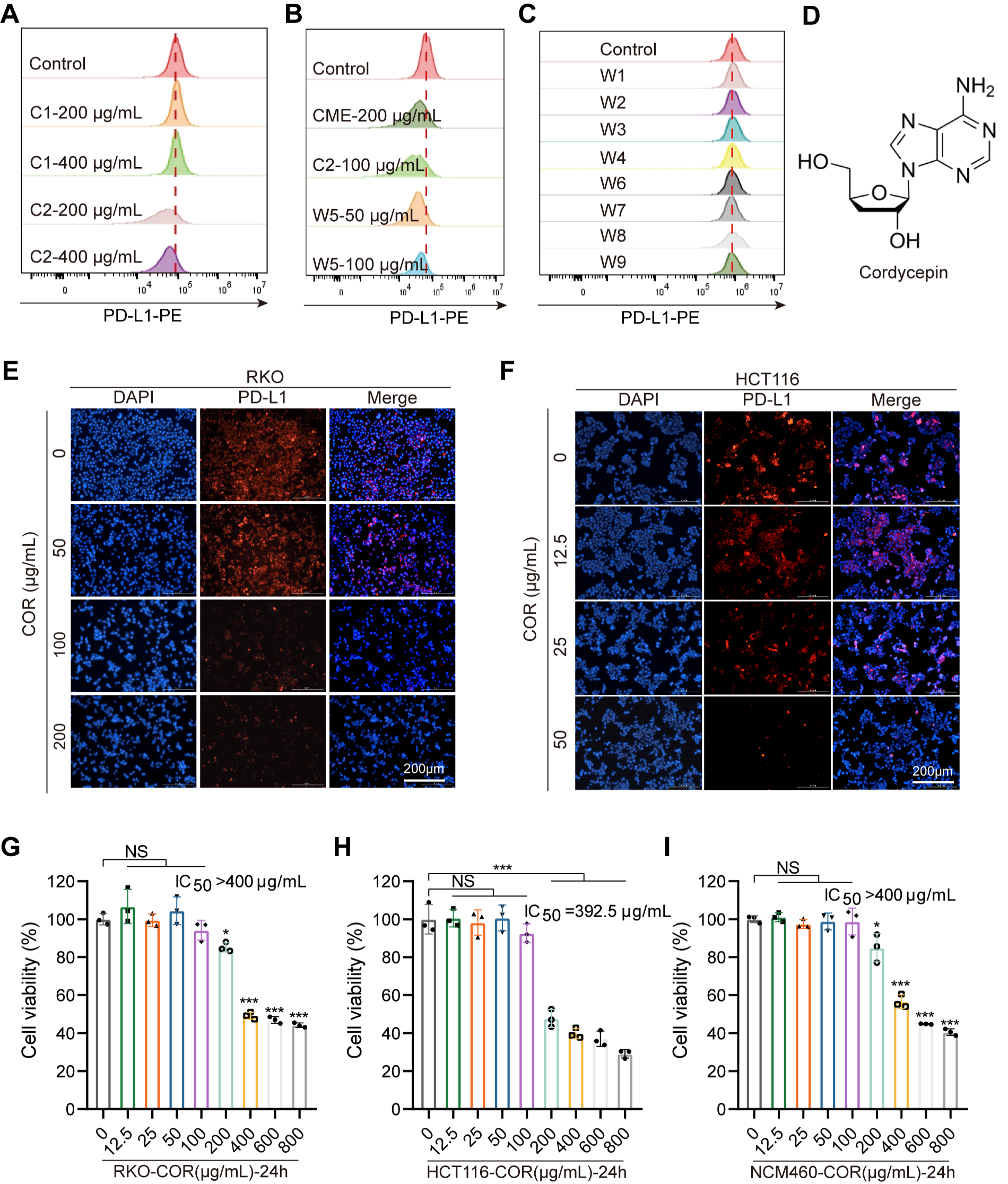


**Figure S3.** **Isolation and confirmation of PD-L1-reducing active ingredients in CME. (A)** The effects of different concentrations of the CME components C1 and C2 on the surface PD-L1 of the RKO cell membrane were detected via flow cytometry. **(B-C)** Detection of the effects of the C2 components W1-W9 on PD-L1 expression on the surface of the RKO cell membrane via flow cytometry. **(D)** The chemical structure of Cordycepin. **(E-F)** An immunofluorescence assay was used to detect the effect of COR on PD-L1 expression on the membrane surface of RKO and HCT116 cells. **(G-I)** A CCK-8 experiment was conducted to detect the cytotoxic effects of COR on RKO cells, HCT116 cells and NCM460 cells at effective concentrations. Statistical differences were determined via Student’s t test. **p* < 0.05; ***p* < 0.01; ****p* < 0.001; NS, not significant.


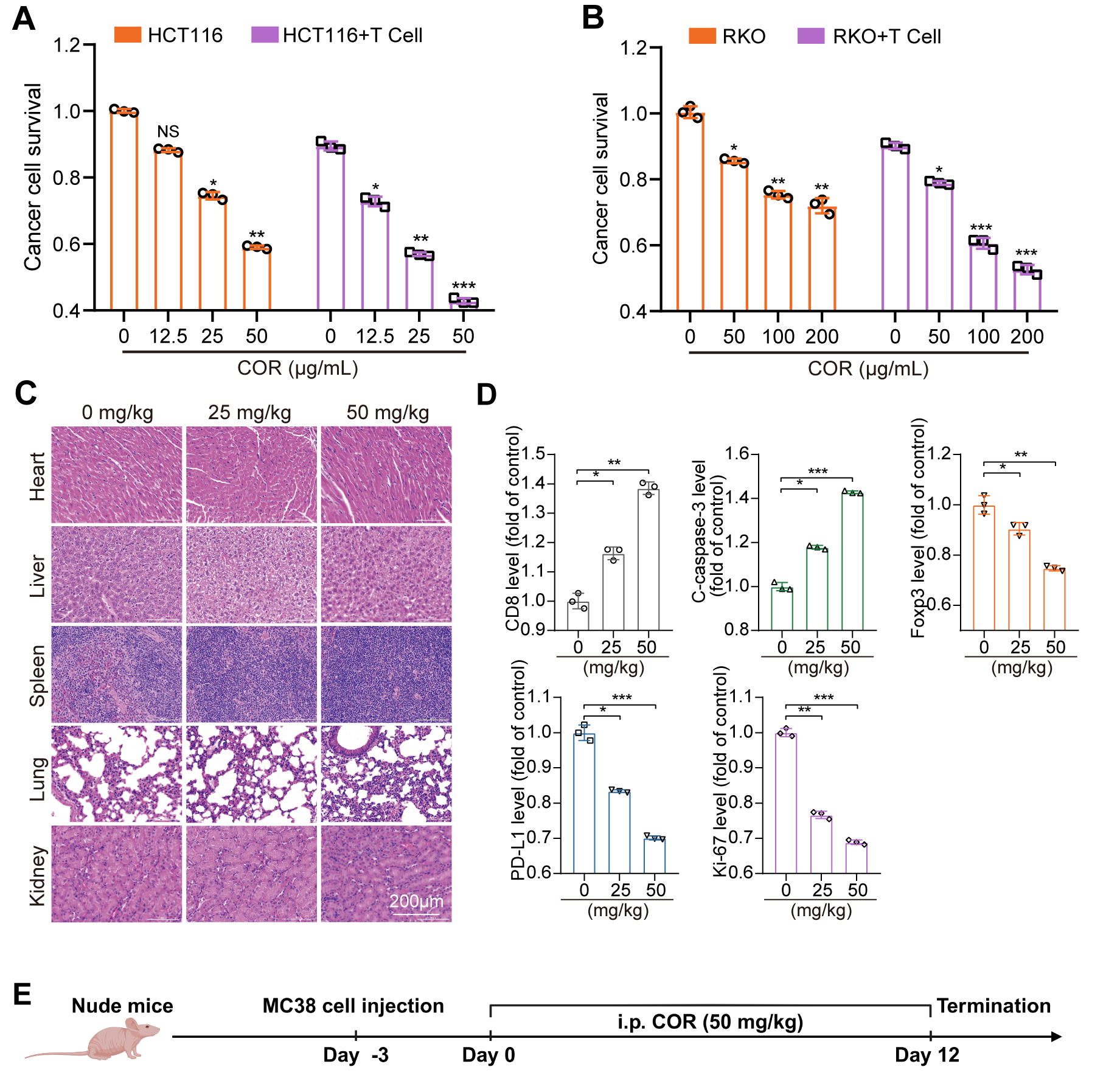


**Figure S4. The killing effect of COR on cancer cells in vitro and in vivo. (A-B)** Analysis of COR-mediated promotion of the killing ability of Jurkat cells against RKO cells and HCT116 cells. **(C)** H&E staining of major organs in C57BL/6J mice in the control group or COR. **(D)** Results of IHC staining for CD8, cleaved cysteine protease-3, FOXP3, PD-L1, and Ki-67. **(E)** Flowchart of the animal experiments involving immunodeficient mice (female). Statistical differences were determined via Student’s t test. NS, not significant, ^#^*p*< 0.05 compared with the RKO or HCT116 DMSO group; **p* < 0.05; ***p* < 0.01; ****p* < 0.001 compared with the RKO + Jurkat cell or HCT116 + Jurkat cell group.


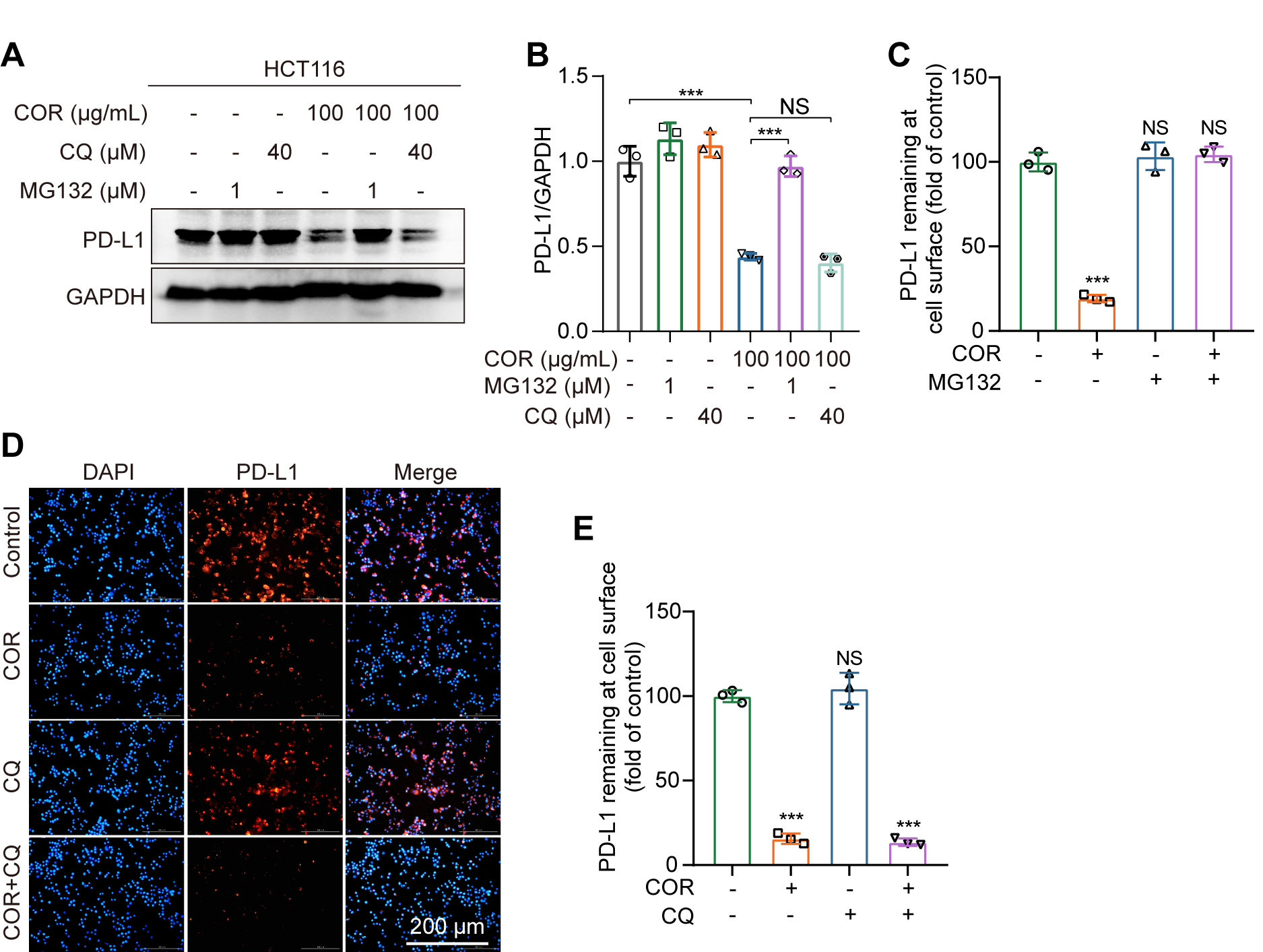


**Figure S5. The ubiquitin proteasome inhibitor MG132 can reverse the degradation of PD-L1 by COR. (A-B)** Effects of the combination of COR, MG132, and CQ inhibitors on PD-L1 levels in HCT116 cells. **(C-E)** An immunofluorescence assay was used to observe the changes in PD-L1 protein expression on the surface of HCT116 cell membranes when COR was used in combination with MG132 or CQ. Statistical differences were determined by Student’s t test. **p* < 0.05; ***p* < 0.01; ****p* < 0.001; NS, not significant.


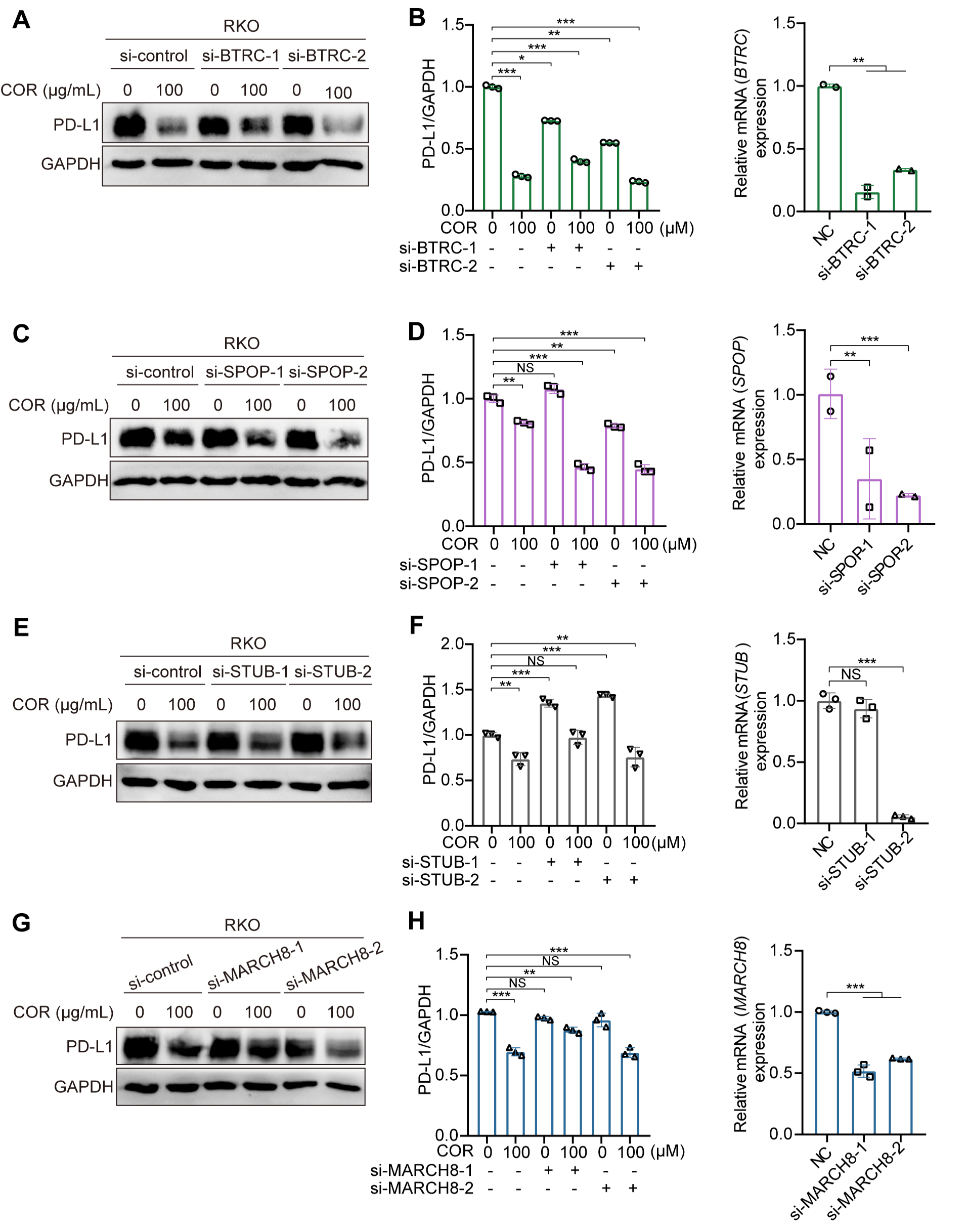


**Figure S6.** **The effect of COR and E3 ligase co treatment on PD-L1 expression level. (A-H)** RKO cells were treated with siRNAs targeting BTRC, SPOP, STUB, MARCH8 or control siRNA, followed by treatment with COR, and changes in PD-L1 expression were detected via protein immunoblotting. Quantitative RT-PCR was used to detect the knockdown efficiency of the siRNAs, and statistical analysis of the immunoblot results was performed. Statistical significance is denoted as **p* < 0.05, ***p* < 0.01, and ****p* < 0.001.


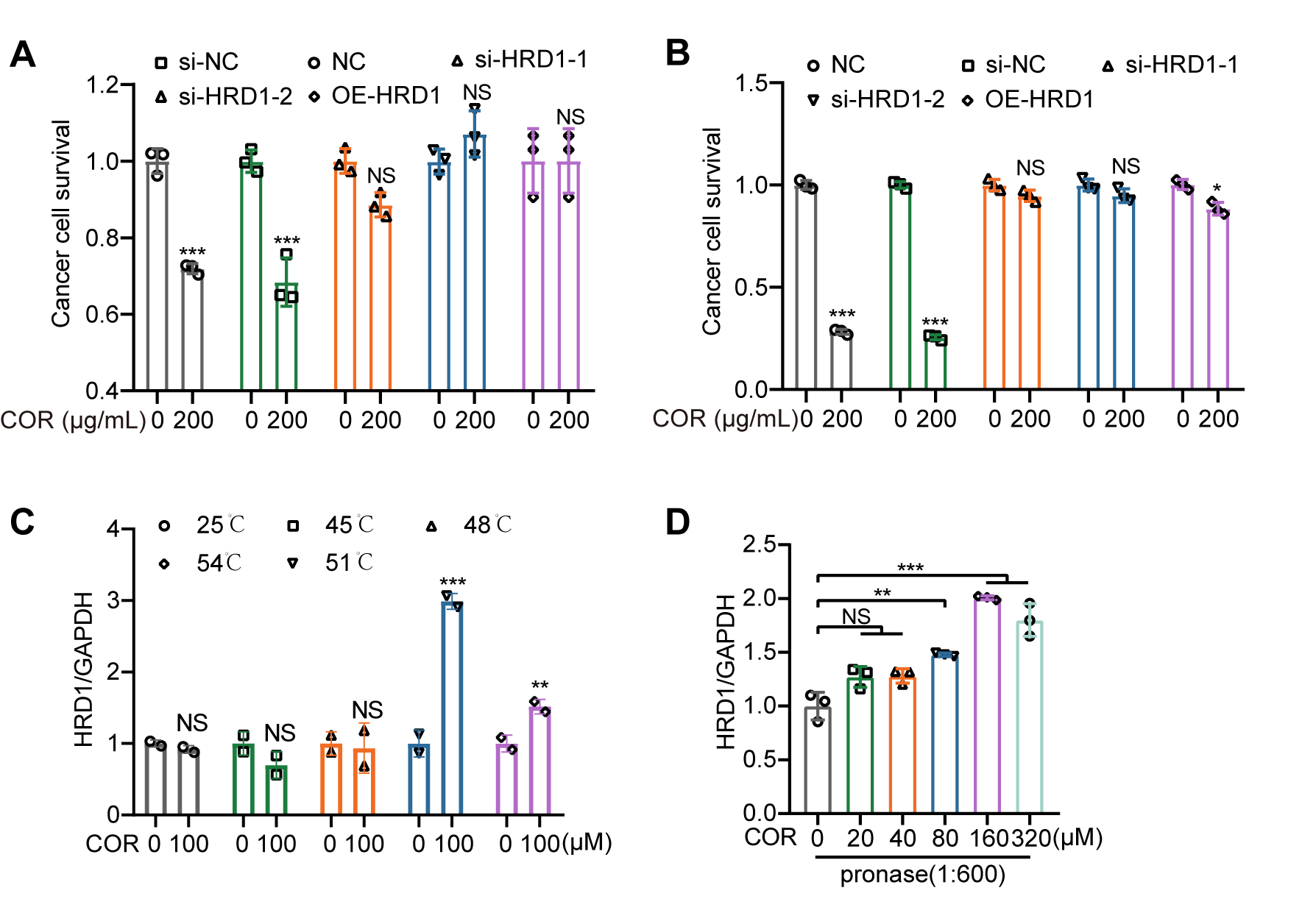


**Figure S7.** **The interaction relationship between COR and HRD1.** **(A and B)** Statistical chart of T cell killing under the action of COR and HRD1. **(C)** Statistical chart of CETSA experiment. **(D)** Statistical chart of streptavidin experiment. The statistical significance is expressed as **p*<0.05, ** *p*<0.01, and ****p*<0.001.


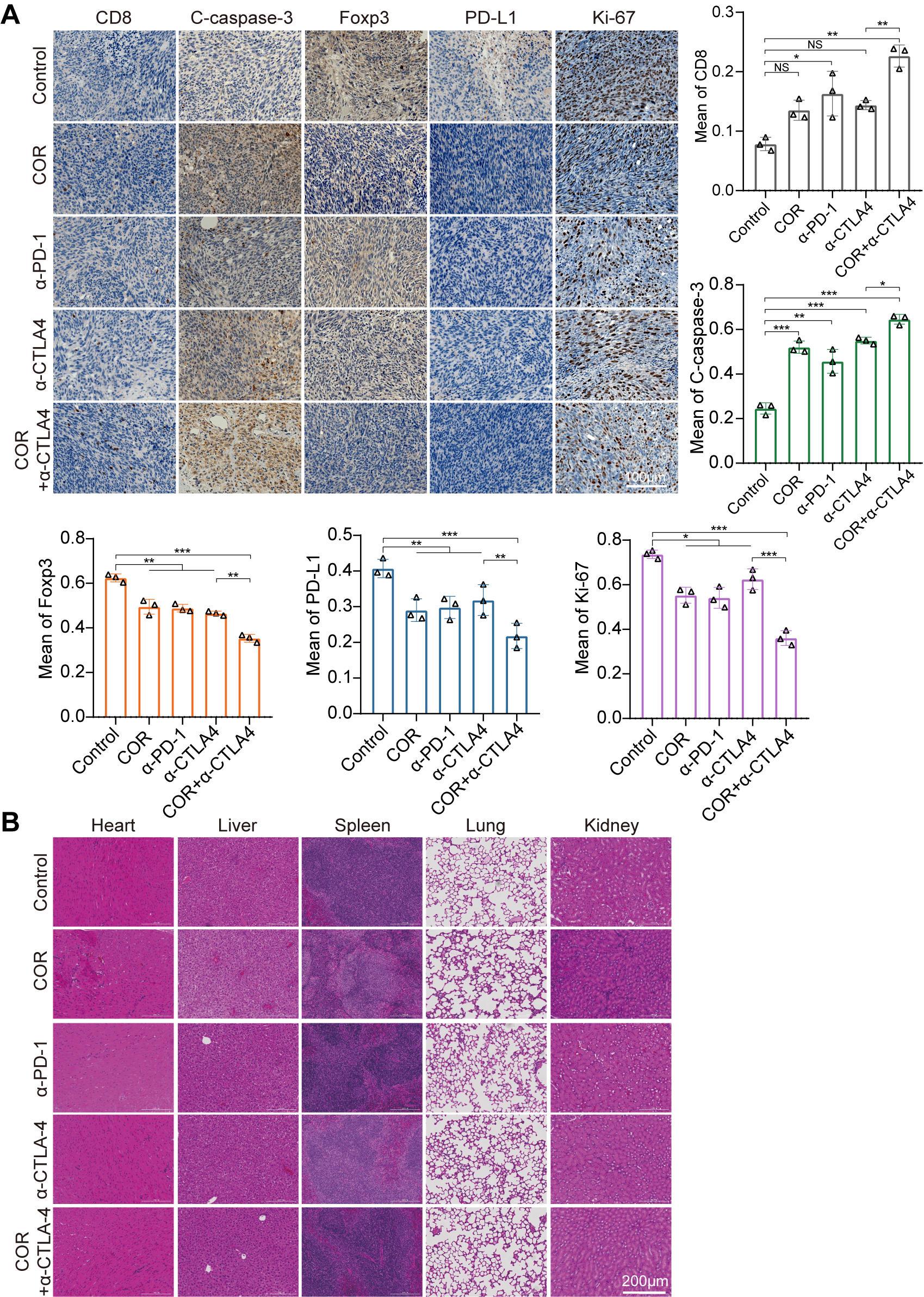


**Figure S8. The combined application of COR and anti-CTLA4 has an impact on the immune microenvironment.** **(A)** Results of IHC staining for CD8, cleaved cysteine protease-3, FOXP3, PD-L1, and Ki-67. (Scale bar = 100 μm). **(B)** Hematoxylin and eosin staining of major organs in C57BL/6J mice treated with control or COR. (Scale bar = 200 μm) The data shown are mean ± standard error (SEM) **p*<0.05, ** *p*<0.01, and *** *p*<0.001.


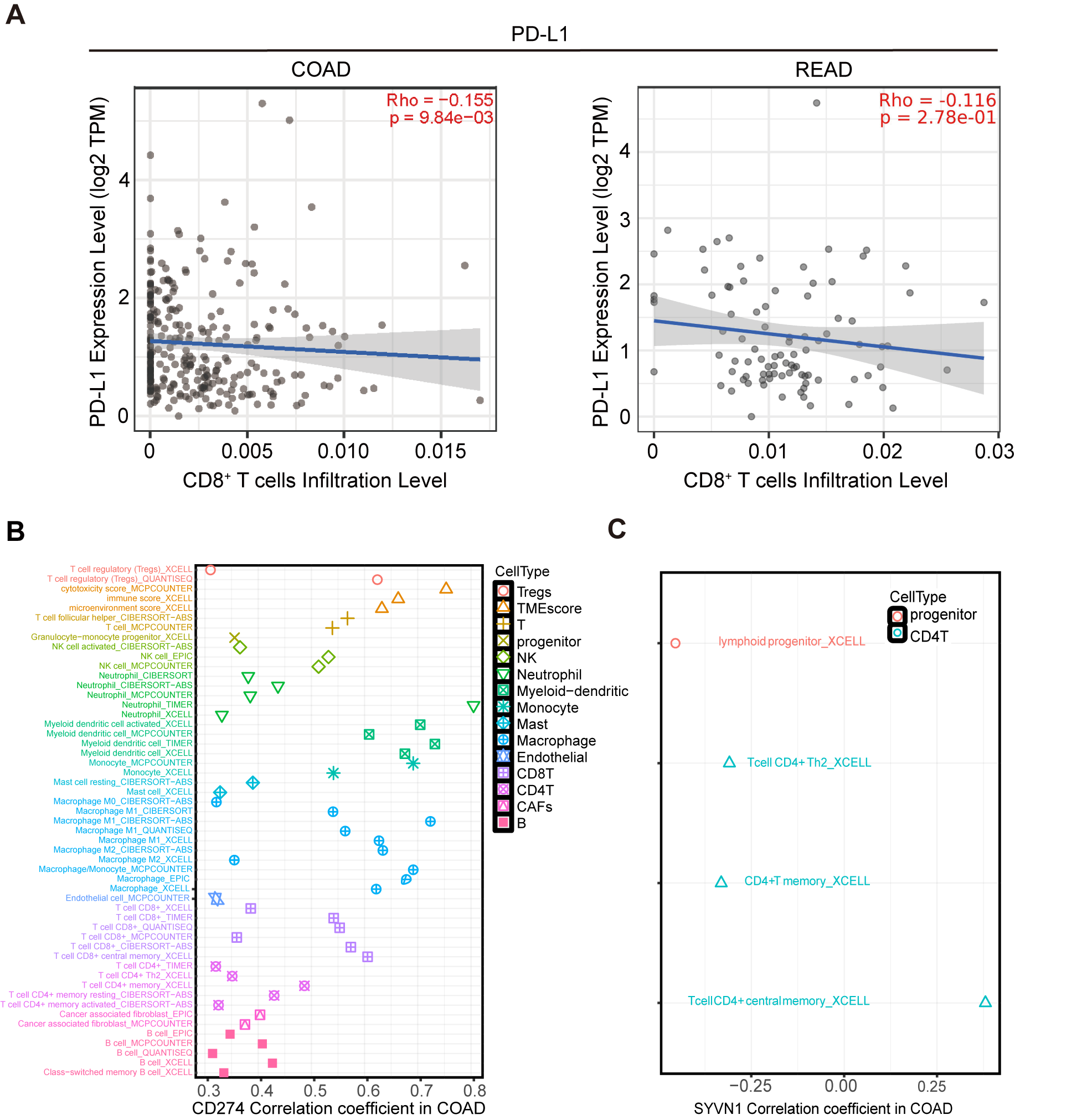


**Figure S9.** **Clinical relevance of HRD1 in the treatment of patients with colon cancer. (A)** Visualization of the correlation between PD-L1 expression and infiltrating CD8^+^ T cells in COAD or READ patients via scatter plots generated with TIMER 2.0. **(B and C)** Correlations between the levels of different immune cell types and PD-L1 expression in patients with COAD.


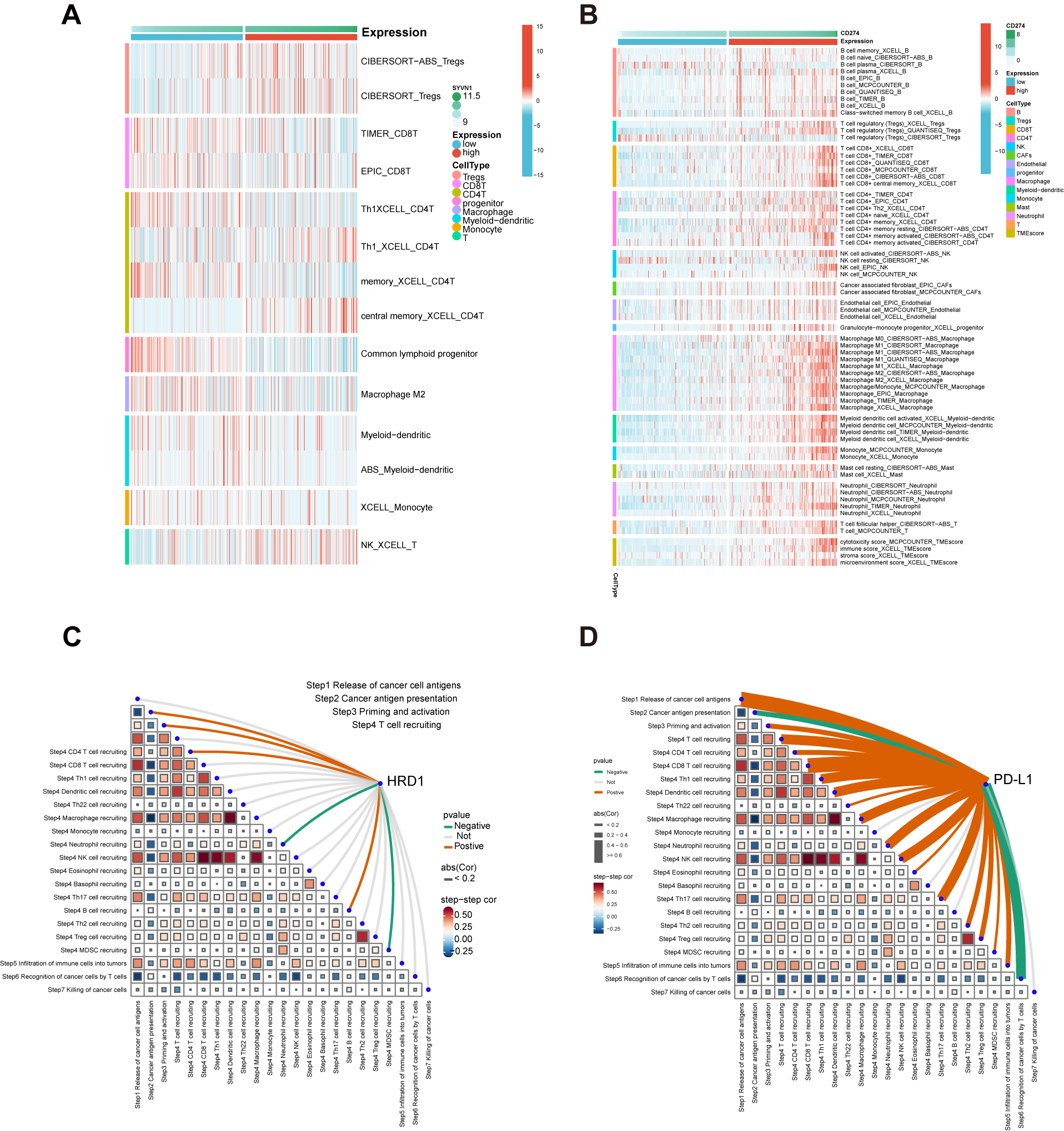


**Figure S10.** **The clinical correlation between HRD1 and cancer treatment.** **(A and B)** Correlations between the levels of different immune cell types and PD-L1 expression in patients with COAD. **(C and D)** Correlations between PD-L1 expression and steps of the cancer immune cycle in patients with COAD.

**Supplementary Table 1. Antibody**

| Name | Source | Catalog No. |
| --- | --- | --- |
| Anti-PD-L1 | Abcam | ab203103 |
| Anti-PD-L1 | Proteintech | 66248-1-Ig |
| Anti-Ubiquitin | Abcam | Ab7245 |
| Anti-HRD1 | Proteintech | 13473-1-AP |
| Anti-GAPDH | Proteintech | 60004-1-Ig |
| PE anti-human CD274 | Biolegend | 329706 |
| Anti-mouse PD-1 (CD279) | Invivogen | BE0146 |
| Anti-mouseCTLA4 (CD152) | Invivogen | BP0032 |
| PE anti-human CD274 | Biolegend | 329706 |
| PE anti-mouse CD274 | Biolegend | 124307 |
| PE anti-mouse CD25 | Biolegend | 101904 |
| Anti-Mouse CD4, PE-Cy7 | Multi Sciences | 70-F2100405/2-100 |
| Anti-Mouse Ly-6G(Gr-1), FITC | Multi Sciences | 70-F21LY6G01100 |
| Anti-Mouse Foxp3, APC | Multi Sciences | 70-F21FP303-100 |
| Anti-Mouse CD3ε, APC-Cy7 | Multi Sciences | 70-F21003A06-100 |
| Anti-Mouse CD8α, PerCP-Cy5.5 | Multi Sciences | 70-F2100804-100 |
| Anti-Human/Mouse CD11b, mFluor 450 | Multi Sciences | 70-F41011b07-100 |
| Alexa Fluor® 700 anti-human/mouse Granzyme B Recombinant Antibody | Biolegend | 372222 |

**Supplementary Table 2. Primers for qRT‒PCR**

| Name | Primers: forward primers (F), reverse primers (R) |
| --- | --- |
| PD-L1 | Forward: 5′-TCACTTGGTAATTCTGGGAGC-3′  Reverse: 5′-CTTTGAGTTTGTATCTTGGATGCC-3′ |
| β-actin | Forward: 5′- AGTGACCAGGCAGAAGATGC-3′  Reverse: 5′- CACGTACTCCATCGCTGACA -3′ |
| HRD1 | Forward: 5’-TGCGTAACATCCACACACTG-3’  Reverse: 5’-AGGCTAAACCTTCTGCCTTCA-3’ |
| SPOP | Forward: 5’-CCTGGAGCGCTTAAAGGTCA-3’  Reverse: 5’-AAAGGGAACACAGTGACGCA-3’ |
| MARCH8 | Forward: 5′-AGTGACATTCCACGTCATTGC-3′  Reverse: 5′-GATCTCCTCAGCAGTACGGTC-3′ |
| BTRC | Forward: 5’-TGGCTCATCTGACAACACTATC-3’  Reverse: 5’-CGAATACAACGCACCAATTCC-3’ |
| STUB1 | Forward: 5′-TCAAGGAGCAGGGCAATCGTCT-3′  Reverse: 5′-GCATCTTCAGGTAGCACAAGGC-3′ |

**Supplementary Table 3. siRNA sequences used for knocking down the indicated proteins**

| Name | Sequence |
| --- | --- |
| si-HRD1#1 | 5′-UGUCUGGCCUUCACCGUUU-3′ |
| si-HRD1#2 | 5′-CCAAGAGACUGCCCUGCAA-3′ |
| si-SPOP#1 | 5′-CACAAGGCUAUCUUAGCAGCU-3′ |
| si-SPOP#2 | 5′-CUCCUACAUGUGGACCAUCAA-3′ |
| si-BTRC#1 | 5′-GCGUUGUAUUCGAUUUGAUAA-3′ |
| si-BTRC#2 | 5′-GCUGAACUUGUGUGCAAGGAA-3′ |
| si-MARCH8#1 | 5’-GGACATTTCATGAGT CATT-3’ |
| si-MARCH8#2 | 5’-GGAAGAGACTCAAGGCCTA-3’ |
| si-STUB1#1 | 5′-GCAGUCUGUGAAGGCGCACUU-3′ |
| si-STUB2#2 | 5′-CCCAAGUUCUGCUGUUGGACU-3′ |
